# Sulfonamide‐Based 2‐Amino‐1,3,4‐Oxadiazole Derivatives as Dual Cholinesterase and Carbonic Anhydrase Inhibitors: Design, Synthesis, Kinetic Characterization, Molecular Modeling, and ADMET Evaluation

**DOI:** 10.1002/ddr.70375

**Published:** 2026-08-25

**Authors:** Gülnur Arslan Karahan, Başak Gökçe, Muhammed Tilahun Muhammed, Emre Kadir Ayan, Azime Berna Özçelik

**Affiliations:** ^1^ Department of Pharmaceutical Chemistry, Faculty of Pharmacy Suleyman Demirel University Isparta Türkiye; ^2^ Department of Biochemistry, Faculty of Pharmacy Suleyman Demirel University Isparta Türkiye; ^3^ Department of Pharmaceutical Chemistry, Faculty of Pharmacy Gazi University Ankara Türkiye

**Keywords:** Alzheimer's disease, benzazolone, carbonic anhydrase, cholinesterase, molecular modeling, oxadiazole

## Abstract

In this study, 12 novel sulfonamide derivatives incorporating 2‐amino‐1,3,4‐oxadiazole and 1,3‐benzazol‐2(3*H*)‐one scaffolds were synthesized and structurally characterized using ^1^H NMR, ^13^C NMR, and LC–MS analyses. Their inhibitory activities were evaluated against acetylcholinesterase (AChE), butyrylcholinesterase (BChE), carbonic anhydrase I and II (CA I and CA II), and α‐glucosidase. Owing to their potent inhibitory profiles, enzyme kinetic studies were performed to determine inhibition constants (*Ki*) and inhibition mechanisms. The synthesized compounds exhibited potent nanomolar cholinesterase inhibition. Compound **5b** was identified as the most active AChE inhibitor (IC_50_ = 32.6 ± 2.6 nM; *Kᵢ* = 7.1 ± 0.6 nM), acting through a competitive inhibition mechanism, whereas compound **5g** showed the strongest BChE inhibition (IC_50_ = 34.6 ± 2.8 nM; *Kᵢ* = 8.4 ± 0.8 nM) with a mixed‐type inhibition profile. The derivatives also displayed significant carbonic anhydrase inhibition, with compound **6d** emerging as the most potent inhibitor, particularly against CA II (IC_50_ = 0.9 ± 0.06 μM; *Kᵢ* = 0.43 ± 0.037 μM), while exhibiting competitive inhibition and strong activity against both CA isoforms. In contrast, α‐glucosidase inhibition was generally weak, with compound **5d** showing the highest activity (IC_50_ = 250.5 ± 21.32 μM). Molecular docking studies revealed favorable binding interactions within the catalytic sites of cholinesterase and carbonic anhydrase enzymes, while 200 ns length molecular dynamics simulations confirmed the stability of representative protein–ligand complexes. In silico ADMET and drug‐likeness predictions using SwissADME and pkCSM indicated generally acceptable oral drug‐likeness. Compound **6c** exhibited the most balanced predicted pharmacokinetic profile, whereas **6d**, despite its excellent enzyme inhibition, showed potential limitations associated with higher polarity, limited predicted blood–brain barrier penetration, and predicted toxicity risks. Overall, these findings identify the synthesized sulfonamide derivatives as promising scaffolds for the further development of multifunctional cholinesterase and carbonic anhydrase inhibitors.

## 1 Introduction

Alzheimer's disease (AD) is a progressive and multifactorial neurodegenerative disorder characterized by progressive cognitive decline and memory impairment, arising from the interplay of several pathological mechanisms, including cholinergic dysfunction, oxidative stress, protein aggregation, neuroinflammation, metabolic dysregulation, and impaired cellular homeostasis (Huang et al. 2016; Seo and Holtzman 2024). Among these mechanisms, impairment of the cholinergic neurotransmission system remains one of the most extensively validated therapeutic targets. Acetylcholinesterase (AChE) and butyrylcholinesterase (BChE), the enzymes responsible for the hydrolysis of acetylcholine (ACh), play central roles in regulating synaptic cholinergic signaling. Their inhibition increases ACh availability within the synaptic cleft and continues to represent the main symptomatic therapeutic strategy for AD (Marucci et al. 2021). While AChE has traditionally been considered the primary therapeutic target, increasing evidence indicates that BChE activity progressively increases during disease progression and partially compensates for declining AChE function, highlighting the therapeutic value of dual cholinesterase inhibition throughout different stages of AD (H. Ferreira‐Vieira et al. 2016; Khan et al. 2023).

In addition to cholinergic dysfunction, disturbances in cerebral pH regulation and metabolic homeostasis have also been implicated in neurodegenerative processes. Carbonic anhydrases (CAs) are zinc‐dependent metalloenzymes that catalyze the reversible hydration of carbon dioxide and play indispensable roles in pH regulation, electrolyte transport, respiration, and cellular metabolism (Supuran 2008, 2016). Among the various isoforms, human carbonic anhydrase I and II (hCA I and hCA II) have attracted considerable attention because of their involvement in cerebral pH regulation, cerebrospinal fluid secretion, oxidative stress, neuroinflammation, and several neurological disorders, including epilepsy and neurodegeneration (Bozdag et al. 2014; Küçükoğlu et al. 2022; Kumar et al. 2022). Accordingly, modulation of CA activity has emerged as a complementary neuroprotective strategy that may enhance the therapeutic efficacy of cholinesterase inhibitors in AD.

**FIGURE 1:**
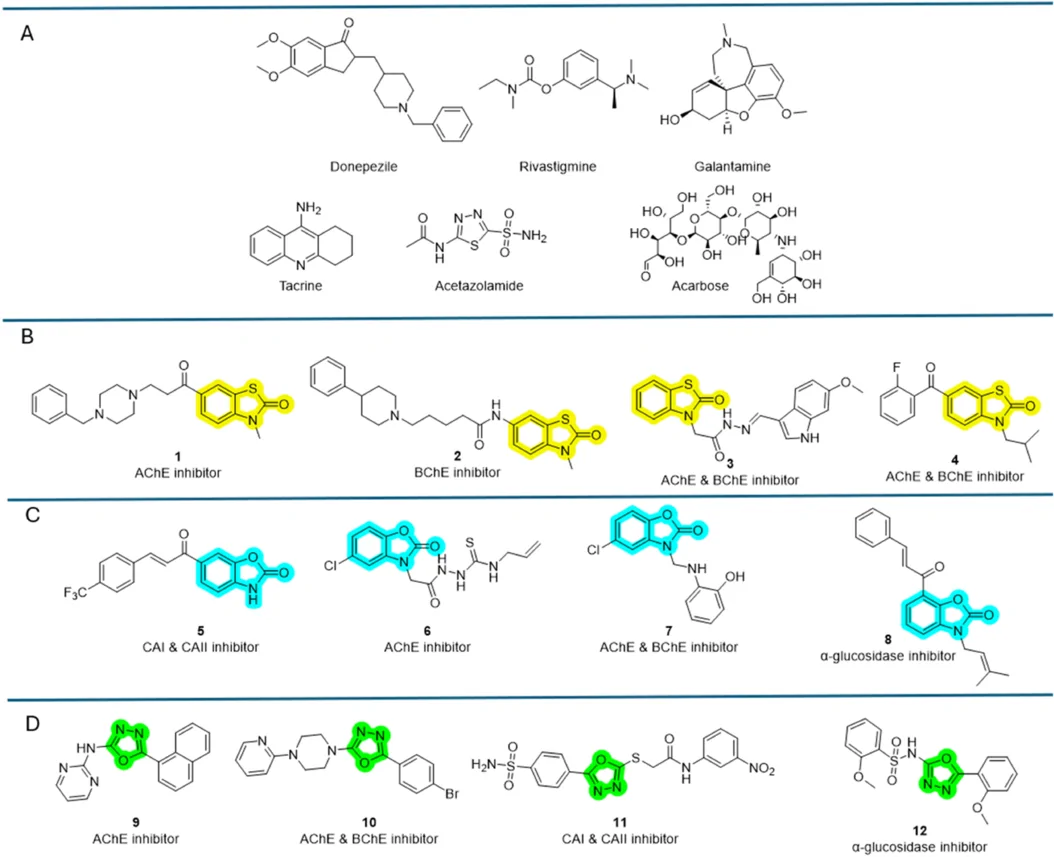
Standard inhibitors (A), bioactive benzothiazolones (B), bioactive benzoxazolones (C), bioactive oxadiazoles (D).

Metabolic dysfunction is increasingly recognized as another hallmark contributing to AD pathogenesis. Epidemiological, clinical, and mechanistic studies have established a close association between type 2 diabetes mellitus (T2DM) and AD, leading to the concept of AD as “type 3 diabetes,” in which insulin resistance, impaired cerebral glucose metabolism, and chronic hyperglycemia collectively accelerate neurodegenerative cascades and cognitive decline (Arnold et al. 2018; De la Monte and Wands 2008; Hull et al. 2018). In this context, inhibition of α‐glucosidase, an enzyme responsible for the hydrolysis of dietary carbohydrates into absorbable monosaccharides, represents an established therapeutic strategy for controlling postprandial hyperglycemia and may indirectly attenuate diabetes‐associated neurodegeneration (Dowarah and Singh 2020; Rojas‐Carranza et al. 2018). The development of multifunctional molecules capable of simultaneously modulating several disease‐relevant targets has therefore emerged as a promising strategy for the treatment of complex disorders such as AD. Representative clinical inhibitors of AChE, BChE, CA I, CA II, and α‐glucosidase are presented in Figure 1A.

The 1,3‐benzazol‐2(3*H*)‐one scaffold, including its benzothiazolone and benzoxazolone derivatives, represents a privileged heterocyclic framework that has attracted considerable attention in medicinal chemistry because of its broad pharmacological applicability. Owing to its balanced lipophilic–hydrophilic character, structural rigidity, and favorable hydrogen‐bonding capability, this scaffold provides an excellent platform for interacting with diverse biological targets (Poupaert et al. 2005). Likewise, the 1,3,4‐oxadiazole ring is a well‐established bioisostere of ester and amide functionalities that enhances metabolic stability, improves pharmacokinetic properties, and facilitates efficient target recognition through hydrogen‐bonding and dipole–dipole interactions (Boström et al. 2012). In addition, benzenesulfonamide constitutes the pharmacophoric core of classical carbonic anhydrase inhibitors because of its strong coordination with the catalytic Zn^2+^ ion within the enzyme active site (Krishnamurthy et al. 2008; Supuran 2008; Supuran et al. 2003). The complementary structural and physicochemical characteristics of these three privileged pharmacophores make them attractive building blocks for the rational design of multifunctional ligands.

Representative benzothiazolone derivatives (Compounds **1–4**, Figure 1B) have been reported as potent cholinesterase inhibitors with nanomolar to low‐micromolar inhibitory activities (Alagöz et al. 2022; Alagöz et al. 2022; Erdogan et al. 2021). Likewise, benzoxazolone derivatives (Compounds **5–8**, Figure 1C) exhibited promising inhibitory activities against cholinesterases and carbonic anhydrases (Bilginer et al. 2019; Koçak Aslan et al. 2023; Uysal et al. 2018; Zhang et al. 2022), whereas oxadiazole‐containing compounds (Compounds **9–12**, Figure 1D) demonstrated potent inhibition of α‐glucosidase and cholinesterases together with additional neuroprotective properties (Sharma et al. 2020; Tripathi et al. 2019; Tripathi et al. 2019; Ullah et al. 2023).

Despite the considerable progress achieved with benzazolone‐, 1,3,4‐oxadiazole‐, and sulfonamide‐based bioactive compounds, these pharmacophores have predominantly been investigated as independent structural motifs or in combination with different molecular frameworks. Their rational integration into a single hybrid scaffold for the simultaneous modulation of cholinesterases, carbonic anhydrases, and α‐glucosidase has received limited attention. Therefore, combining these complementary pharmacophores represents a rational strategy for the development of multifunctional ligands targeting the multifactorial pathology of Alzheimer's disease and its associated metabolic dysfunction.

**FIGURE 2:**
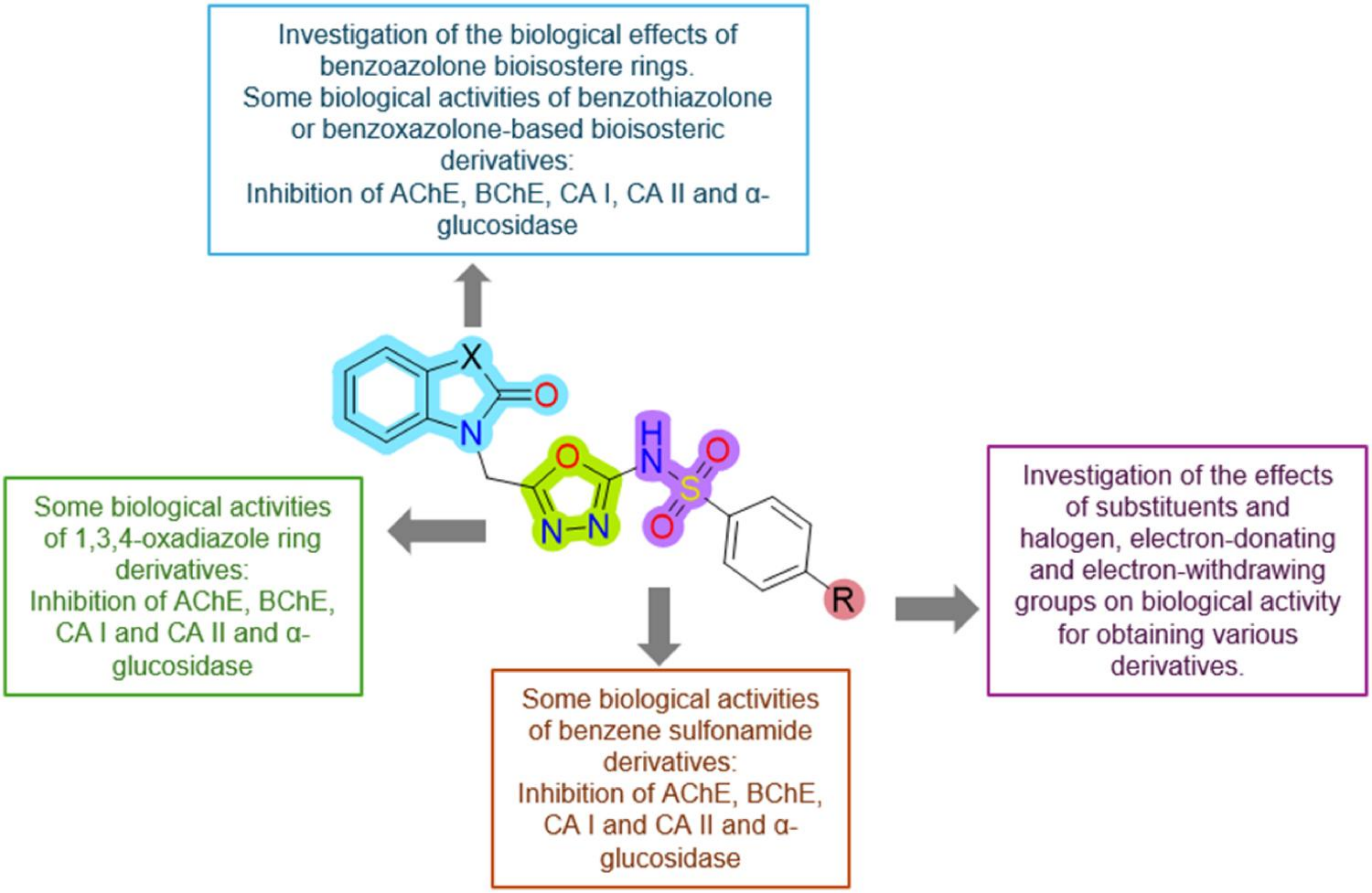
Rational design of compounds.

It should be emphasized that the final target compounds (**5a–g** and **6a–e**) represent novel chemical entities that, to the best of our knowledge, have not been previously reported in the literature. Although the individual pharmacophores, namely 1,3‐benzazol‐2(3*H*)‐one, 2‐amino‐1,3,4‐oxadiazole, and benzenesulfonamide, are well established in medicinal chemistry, they have been integrated for the first time into the present molecular framework. Accordingly, the novelty of this work lies in the design and first synthesis of these original hybrid molecules together with their comprehensive biological, kinetic, molecular docking, molecular Dynamics simulation, and ADMET evaluation. Based on this rationale (Figure 2), a series of sulfonamide derivatives bearing the 3‐[(5‐amino‐1,3,4‐oxadiazol‐2‐yl)methyl]−1,3‐benzazol‐2(3*H*)‐one core was synthesized and evaluated against AChE, BChE, hCA I, hCA II, and α‐glucosidase to investigate their inhibitory activities, binding mechanisms, and drug‐like properties.

## 2 Results and Discussion

### 2.1 Chemistry

**FIGURE 3:**
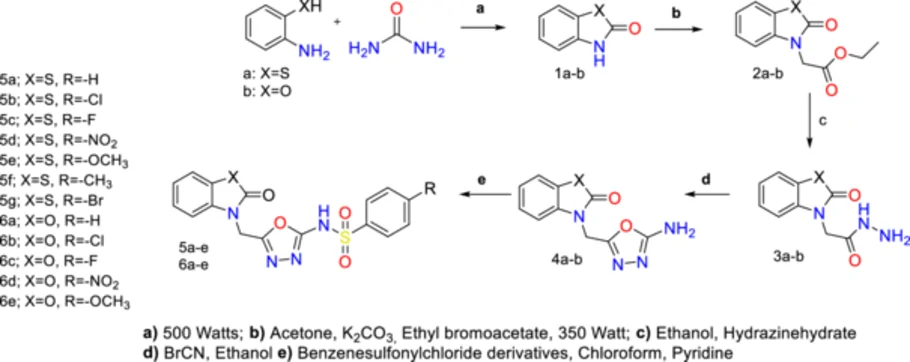
General synthesis scheme.

The target compounds (**5a–g** and **6a–e**) were synthesized through a multistep and modular synthetic route starting from benzothiazolone‐ and benzoxazolone‐based precursors (**1a–b**), as outlined in Figure 3. Initially, ester derivatives (**2a–b**) were obtained via alkylation reactions, followed by conversion to the corresponding hydrazides (**3a–b**). Subsequent cyclization of the hydrazide intermediates afforded the key 2‐amino‐1,3,4‐oxadiazole derivatives (**4a–b**), which were finally reacted with various substituted benzenesulfonyl chlorides to furnish the desired sulfonamide derivatives (**5a–g** and **6a–e**).

Prior to biological evaluation, the purity and structural integrity of all synthesized compounds were confirmed by TLC, LC–MS, and ^1^H/^13^C NMR spectroscopy. The analytical data were fully consistent with the proposed structures. All compounds were recorded in DMSO‐*d*_6_ using 500 MHz (^1^H NMR) and 125 MHz (^13^C NMR) spectrometers.

In the ^1^H NMR spectra, the methylene protons were observed as singlets at 5.20–5.31 ppm, whereas no distinct resonance corresponding to the sulfonamide NH proton was observed, most likely because of rapid proton exchange under the experimental conditions. The remaining proton resonances were assigned to the expected aromatic regions.

The ^13^C NMR spectra further supported the proposed structures. The carbonyl carbon of the benzothiazolone ring resonated at 169.35–169.38 ppm, whereas that of the benzoxazolone ring appeared at 157.52–157.74 ppm. The methylene carbon resonated at 37.03–37.29 ppm, while the C‐2 and C‐5 carbons of the 1,3,4‐oxadiazole ring appeared at 153.66–154.65 ppm and 154.13–156.93 ppm, respectively. As expected, fluorinated derivatives exhibited characteristic long‐range ^13^C–^19^F coupling patterns, which further supported their structural assignments. Detailed spectroscopic data (^1^H NMR, ^13^C NMR, and LC–MS) for all final compounds are provided in the Supporting Information.

### 2.2 Biological Evaluation

#### 2.2.1 Cholinesterase Inhibitory Activity and Structure–Activity Relationship (SAR) Studies

Galantamine and rivastigmine were selected as clinically used reference inhibitors because of their well‐established inhibitory profiles against both AChE and BChE. The active site of AChE consists of a deep and narrow catalytic gorge comprising the catalytic triad (Ser203–His447–Glu334), the anionic subsite centered on Trp86, and the peripheral anionic site (PAS), primarily formed by Trp286, Tyr72, and Tyr124. Kinetic analyses demonstrated that all synthesized benzothiazolone (**5a–g**) and benzoxazolone (**6a–e**) derivatives acted as competitive inhibitors of AChE, indicating that these compounds preferentially occupy the catalytic binding gorge and compete with the natural substrate acetylcholine for access to the active site (Table 1) (Lu et al. 2011; Rawat et al. 2024).

**TABLE 1:** Enzyme kinetic parameters of the synthesized compounds for AChE and BChE inhibition.

	AchE			BchE			
Compounds	IC_50_ (nM)	*Kᵢ* (nM)	Inhibition Type	IC_50_ (nM)	*Kᵢ* (nM)	Inhibition Type	SI(*K*_*i*_)^1^
**5a**	96.4 ± 8.5	22.1 ± 1.8	Competitive	92.4 ± 7.4	22.2 ± 1.8	mix	1.00
**5b**	**32.6** ± **2.6**	**7.1** ± **0.6**	**Competitive**	42.7 ± 3.4	10.8 ± 0.9	mix	1.52
**5c**	64.3 ± 5.1	15.7 ± 1.3	Competitive	40.1 ± 3.2	9.7 ± 0.8	competitive	0.62
**5d**	57.2 ± 4.6	13.4 ± 1.1	Competitive	67.6 ± 5.4	16.3 ± 1.3	mix	1.22
**5e**	52.3 ± 4.2	12.8 ± 1.2	Competitive	59.7 ± 4.8	12.6 ± 1.2	mix	0.98
**5f**	73.7 ± 5.9	17.6 ± 1.4	Competitive	77.4 ± 6.2	18.3 ± 1.6	mix	1.04
**5g**	41.8 ± 3.3	9.4 ± 0.9	Competitive	**34.6** ± **2.8**	**8.4** ± **0.8**	**mix**	0.89
**6a**	67.5 ± 5.4	27.9 ± 2.1	Competitive	112.4 ± 9.0	26.7 ± 2.1	mix	0.96
**6b**	39.8 ± 3.2	9.7 ± 0.8	Competitive	49.4 ± 4.0	12.7 ± 1.1	mix	1.31
**6c**	77.7 ± 6.2	18.3 ± 1.5	Competitive	47.3 ± 3.8	11.6 ± 0.9	competitive	0.63
**6d**	67.8 ± 5.4	15.7 ± 1.3	Competitive	78.1 ± 6.2	18.4 ± 1.5	mix	1.17
**6e**	64.7 ± 5.2	15.6 ± 1.2	Competitive	73.1 ± 5.8	16.8 ± 1.3	mix	1.08
**GAL**	1202 ± 29	410 ± 22	Competitive	7215 ± 650	1200 ± 18	Competitive	2.93
**RİV**	254 ± 16	ND		37 ± 3.2	17 ± 1.4	Competitive	ND

*Note:* SI(*K*_*i*_)^**1**^: *Kᵢ* (BChE)/*Kᵢ* (AChE); SI > 1 indicates AChE selectivity, whereas SI < 1 indicates BChE selectivity.

Abbreviations: GAL, galantamin reference drug; ND, Not determined due to time‐dependent inhibition; RİV, rivastigmin reference drug.

**FIGURE 4:**
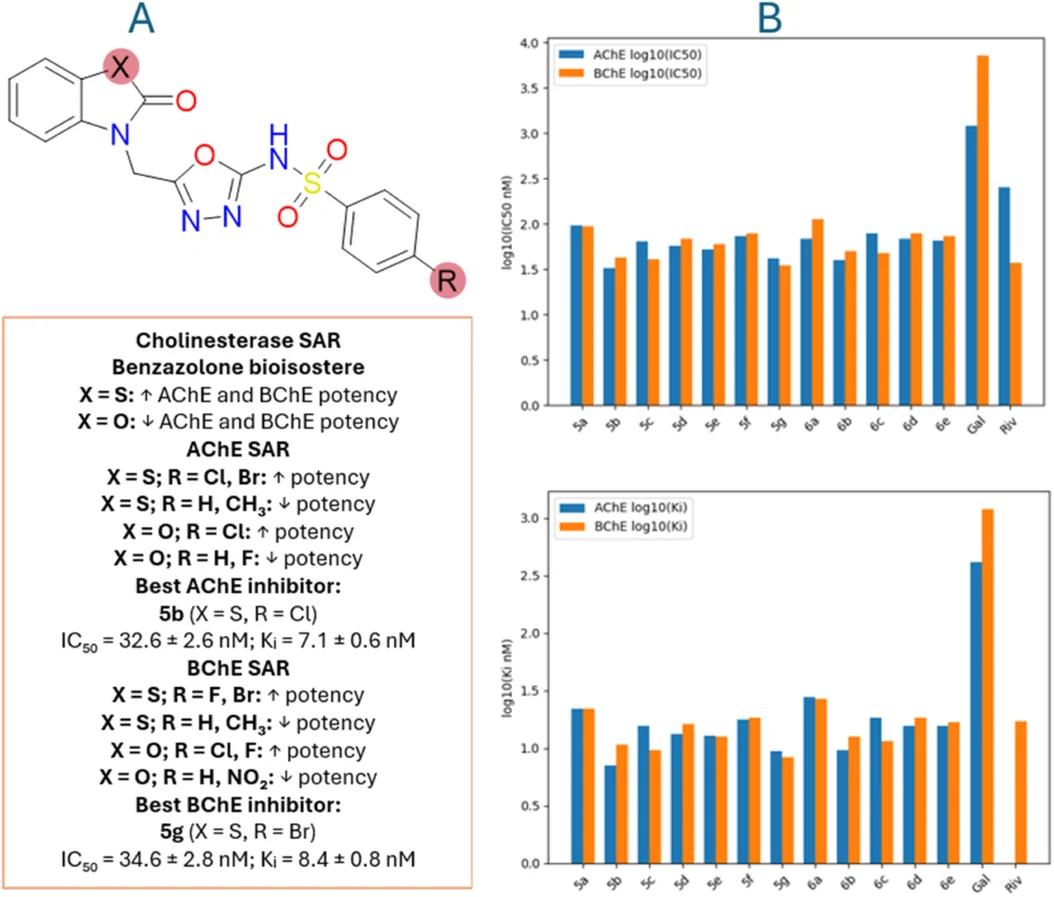
Structure–activity relationship of the synthesized benzothiazolone and benzoxazolone derivatives against AChE and BChE. (A) Summary of the observed SAR trends according to scaffold and aromatic substitution. (B) Comparison of IC_50_ and *Kᵢ* values for AChE and BChE inhibition.

All synthesized compounds exhibited markedly lower IC_50_ values than the reference inhibitors galantamine (IC_50_ = 1202 ± 29 nM) and rivastigmine (IC_50_ = 254 ± 16 nM), demonstrating their remarkable inhibitory potency toward AChE (Table 1). Overall, the benzothiazolone derivatives (**5a–g**) consistently displayed lower IC_50_ and *Kᵢ* values than their corresponding benzoxazolone analogues (**6a–e**). For example, **5b** exhibited higher inhibitory potency (IC_50_ = 32.6 ± 2.6 nM, *Kᵢ* = 7.1 ± 0.6 nM) than its oxygen analogue **6b** (IC_50_ = 39.8 ± 3.2 nM, *Kᵢ* = 9.7 ± 0.8 nM) (Table 1, Figure 4). The consistently superior activity of the sulfur‐containing analogues may be attributed to the higher polarizability, greater hydrophobicity, and larger atomic radius of sulfur relative to oxygen. These properties are expected to favor stronger hydrophobic and π‐sulfur interactions with aromatic residues lining the catalytic gorge, including Trp86 and Phe338, while the slightly longer C–S bond may provide improved geometric complementarity within the narrow active‐site cavity (Lu et al. 2011; Rawat et al. 2024).

The nature of the aromatic substituent also exerted a pronounced influence on AChE inhibitory activity. Among both series, the chloro‐substituted derivatives **5b** and **6b** exhibited the strongest inhibition, suggesting that the moderate electron‐withdrawing character and steric properties of chlorine favor productive enzyme–ligand interactions within the catalytic pocket. In contrast, the unsubstituted analogues **5a** and **6a** displayed the weakest inhibitory activity, emphasizing the importance of aromatic substitution for efficient enzyme recognition. Derivatives bearing methoxy, nitro, and fluoro substituents exhibited intermediate activity, indicating that AChE inhibition is governed not only by electronic effects but also by the combined contributions of steric and hydrophobic interactions.

All synthesized compounds exhibited substantially stronger BChE inhibitory activity than the reference inhibitor galantamine (IC_50_ = 7215 ± 650 nM, *Kᵢ* = 1200 ± 180 nM), whereas several derivatives displayed inhibitory potencies comparable to those of rivastigmine (IC_50_ = 37 ± 3.2 nM, *Kᵢ* = 17 ± 1.4 nM) (Table 1, Figure 4). Consistent with the AChE inhibition profile, the benzothiazolone derivatives (**5a–g**) generally exhibited lower IC_50_ and *Kᵢ* values than the corresponding benzoxazolone analogues (**6a–e**), further supporting the favorable contribution of sulfur to enzyme binding. The higher polarizability of sulfur may strengthen hydrophobic and π‐sulfur interactions with aromatic residues within the BChE active site, thereby contributing to enhanced stabilization of the enzyme–inhibitor complex.

Distinct SAR trends were observed between AChE and BChE. Whereas chloro‐substituted derivatives (**5b** and **6b**) were the most potent inhibitors of AChE, the fluoro‐substituted analogues (**5c** and **6c**) displayed enhanced inhibitory activity toward BChE and were the only compounds that behaved as competitive inhibitors against both cholinesterases. This difference may reflect the larger and more flexible active‐site gorge of BChE, which can better accommodate subtle changes in substituent size and electronic properties. The relatively small steric demand and strong electron‐withdrawing character of fluorine may therefore improve the complementarity of these derivatives within the BChE binding pocket.

Among the synthesized compounds, the brominated derivative **5g** emerged as the most potent BChE inhibitor (IC_50_ = 34.6 ± 2.8 nM, *Kᵢ* = 8.4 ± 0.8 nM). This enhanced activity is consistent with the structural characteristics of the BChE active site, whose enlarged acyl‐binding pocket, containing smaller aliphatic residues such as valine and leucine instead of the aromatic phenylalanines present in AChE, is capable of accommodating bulkier lipophilic substituents more efficiently (Radic et al. 1993). Consequently, the bromine substituent may maximize hydrophobic contacts within this expanded cavity, leading to improved binding affinity.

Kinetic analyses further demonstrated that most synthesized compounds acted as mixed‐type inhibitors of BChE, suggesting simultaneous interactions with both the catalytic site and peripheral binding regions. In contrast, only the fluoro‐substituted derivatives **5c** and **6c** exhibited competitive inhibition toward both cholinesterases, indicating a preferential binding mode centered within the catalytic gorge. Collectively, these findings demonstrate that both the size and electronic characteristics of the aromatic substituent play important roles in determining inhibitory potency and binding mode toward BChE.

Analysis of the selectivity index (SI) values derived from the inhibition constants (*Kᵢ*) revealed that most synthesized compounds displayed a balanced inhibitory profile toward both cholinesterase isoforms, with SI values generally ranging from 0.89 to 1.31, indicating comparable affinity for AChE and BChE (Table 1). Nevertheless, modulation of the aromatic substituent produced discernible changes in isoform selectivity. Among the synthesized derivatives, the chloro‐substituted analogues **5b** (SI = 1.52) and **6b** (SI = 1.31) exhibited the highest preference for AChE, suggesting that the moderate electron‐withdrawing character and steric properties of chlorine favor interactions within the relatively narrow AChE active‐site gorge. In contrast, the fluoro‐substituted derivatives **5c** (SI = 0.62) and **6c** (SI = 0.63) demonstrated a distinct shift toward BChE selectivity, which is consistent with the larger and more conformationally flexible binding cavity of BChE accommodating these analogues more favorably. The nitro‐substituted derivatives **5d** and **6d** exhibited moderate AChE selectivity (SI = 1.22 and 1.17, respectively), whereas the methoxy‐substituted derivatives **5e** and **6e**, together with the methyl‐substituted analogue **5f**, produced SI values close to unity, indicating nearly equivalent inhibition of both cholinesterase isoforms.

Comparison of structurally related sulfur‐ and oxygen‐containing analogue pairs (**5b–6b**, **5c–6c**, **5d–6d**, and **5e–6e**) demonstrated that replacement of sulfur with oxygen exerted only a minor influence on enzyme selectivity. Collectively, these findings indicate that the aromatic substituent is the principal determinant of cholinesterase selectivity within this compound series, whereas the nature of the heteroatom in the benzazole scaffold plays a comparatively secondary role.

Overall, the present findings identify the sulfur‐containing, halogen‐substituted benzothiazolone derivatives as promising scaffolds for the development of dual AChE/BChE inhibitors. In particular, **5b** combined the highest AChE selectivity with nanomolar inhibitory potency, whereas **5g** emerged as the most potent BChE inhibitor. Moreover, **5c** exhibited a well‐balanced dual inhibitory profile together with competitive inhibition against both cholinesterases, highlighting its potential as a lead structure for further optimization in the development of multitarget agents for Alzheimer's disease.

Nevertheless, the proposed structure–activity relationships are derived exclusively from in vitro enzyme inhibition data and should therefore be interpreted together with the molecular docking and molecular dynamics analyses presented below. Furthermore, the calculated SI values primarily reflect differences in binding affinity under defined experimental conditions and do not account for biological variables such as enzyme expression, tissue distribution, cellular uptake, target accessibility, or pharmacokinetic behavior. Consequently, complementary cell‐based and in vivo studies will be required to determine whether the selectivity profiles observed in the present study are maintained under physiologically relevant conditions.

#### 2.2.2 Carbonic Anhydrase Isozymes CA I and CA II Inhibitory Activity and Structure–Activity Relationship (SAR) Studies

Acetazolamide was selected as the reference inhibitor because of its well‐established inhibitory activity against the human carbonic anhydrase isozymes CA I and CA II. All synthesized compounds exhibited stronger inhibition toward hCA II than hCA I, consistent with the isoform selectivity commonly reported for aromatic sulfonamide‐based carbonic anhydrase inhibitors (Gokcen et al. 2016; Tugrak et al. 2021). Although both isoenzymes contain a catalytic Zn^2+^ ion, hCA II possesses a larger and more hydrophobic active‐site cavity enriched with residues such as Val121, Phe131, and Leu198, enabling more favorable accommodation of aromatic inhibitors bearing electron‐withdrawing substituents (Supuran 2008).

**FIGURE 5:**
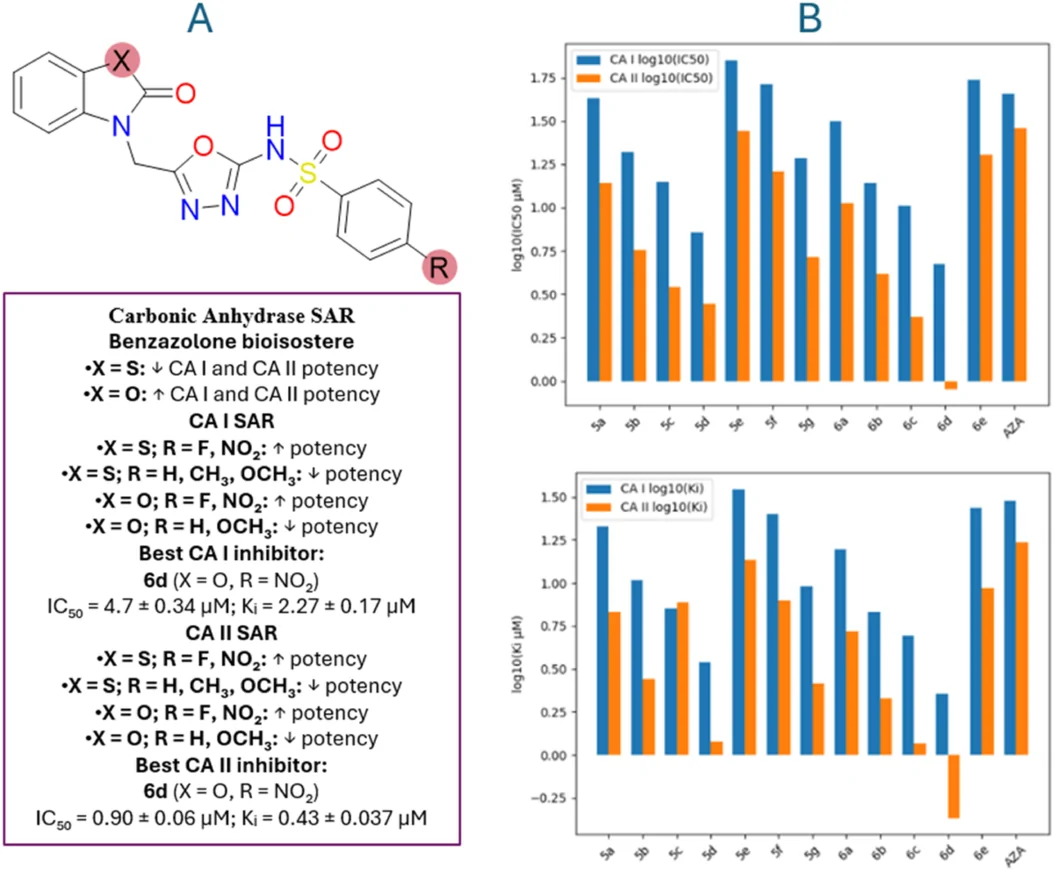
Structure–activity relationship of the synthesized benzothiazolone and benzoxazolone derivatives against carbonic anhydrase isozymes CA I and CA II. (A) Summary of the observed SAR trends according to scaffold and aromatic substitution. (B) Comparison of IC_50_ and *Kᵢ* values for CA I and CA II inhibition.

A clear scaffold‐dependent SAR was observed between the two series. Overall, the benzoxazolone derivatives (**6a–e**) consistently exhibited lower IC_50_ and *Kᵢ* values than the corresponding benzothiazolone analogues (**5a–g**) against both CA isozymes. This trend was particularly evident for the nitro‐substituted pair, where **6 d** (*Kᵢ* = 0.43 ± 0.037 µM for hCA II) displayed substantially higher inhibitory potency than its sulfur analogue **5 d** (Kᵢ = 1.20 ± 0.09 µM). The superior activity of the oxygen‐containing scaffold may be attributed to its smaller steric demand and higher electronegativity, which could facilitate a more favorable orientation within the active site and improve the hydrogen‐bonding network surrounding the catalytic Zn^2+^ ion (Table 2, Figure 5).

**TABLE 2:** Enzyme kinetic parameters of the synthesized compounds for human carbonic anhydrase isozymes CA I and CA II inhibition.

	CAI			CAII			
Compounds	IC_50_ (µM)	*Kᵢ* (µM)	Inhibition Type	IC_50_ (µM)	*Kᵢ* (µM)	Inhibition Type	SI(*K*_*i*_)^2^
**5a**	42.5 ± 5.7	21.26 ± 3.2	competitive	13.86 ± 1.3	6.81 ± 0.7	competitive	3.12
**5b**	21 ± 2.2	10.4 ± 1.2	competitive	5.72 ± 0.45	2.76 ± 0.24	competitive	3.77
**5c**	14 ± 1.4	7.13 ± 0.74	competitive	3.47 ± 0.26	7.72 ± 0.12	competitive	0.92
**5d**	7.2 ± 0.52	3.45 ± 0.24	competitive	2.8 ± 0.23	1.2 ± 0.09	competitive	2.88
**5e**	71 ± 12.31	35.12 ± 6.36	Mixed	27.6 ± 5.48	13.64 ± 2.5	Mixed	2.57
**5f**	51.4 ± 6.2	25.0 ± 3.0	competitive	16.2 ± 1.6	7.9 ± 0.8	competitive	3.16
**5g**	19.3 ± 2.0	9.6 ± 1.0	competitive	5.2 ± 0.4	2.6 ± 0.2	competitive	3.69
**6a**	31.6 ± 3.53	15.74 ± 2.17	competitive	10.62 ± 0.98	5.24 ± 0.48	competitive	3.00
**6b**	13.9 ± 1.25	6.8 ± 0.74	competitive	4.13 ± 0.29	2.14 ± 0.18	competitive	3.18
**6c**	10.2 ± 0.82	4.92 ± 0.54	competitive	2.35 ± 0.21	1.175 ± 0.088	competitive	4.21
**6d**	**4.7** ± **0.34**	**2.27** ± **0.17**	**competitive**	**0.9** ± **0.06**	**0.43** ± **0.037**	**competitive**	5.28
**6e**	54.8 ± 8.6	27.43 ± 5.18	Mixed	20.12 ± 3.4	9.36 ± 1.8	Mixed	2.93
**AZA**	45.14 ± 5.61	30.16 ± 4.61	competitive	28.6 ± 3.7	17.14 ± 2.34	competitive	1.76

*Note*: SI(*Ki*)^2^: *Ki*(CA I)/*Ki*(CA II); SI > 1 indicates CA II selectivity, while SI < 1 indicates CA I selectivity.

Abbreviation: AZA, Acetazolamide reference drug.

Substituent effects also played a decisive role in carbonic anhydrase inhibition. For both scaffold series, the general activity trend followed –NO_2_ > –F ≈ –Cl > –H ≫ –OCH_3_, highlighting the importance of electron‐withdrawing substituents for effective enzyme inhibition. Among all synthesized compounds, the nitro‐substituted derivative **6d** was the most potent inhibitor of both isoforms and exhibited approximately 40‐fold greater hCA II inhibitory potency than acetazolamide based on *Kᵢ* values (Table 2, Figure 5). The strong electron‐withdrawing character of the nitro group is expected to enhance the acidity of the sulfonamide NH, thereby facilitating deprotonation and more effective coordination of the sulfonamide nitrogen to the catalytic Zn^2+^ ion, which is the principal interaction responsible for carbonic anhydrase inhibition.

Fluoro‐ and chloro‐substituted derivatives also exhibited favorable inhibitory activity, with the fluoro analogues generally showing slightly lower IC_50_ and *Kᵢ* values than the corresponding chloro derivatives. This observation is consistent with previous studies demonstrating that fluorine substitution can improve binding affinity in heterocyclic sulfonamide‐based carbonic anhydrase inhibitors through favorable electronic and steric effects (Alım et al. 2020; Gokcen et al. 2016). In contrast, the methoxy‐substituted derivatives **5e** and **6e** showed the weakest inhibitory activity and were the only compounds exhibiting mixed‐type inhibition. The bulky electron‐donating methoxy group may reduce optimal positioning of the sulfonamide moiety within the catalytic pocket, thereby weakening interactions with the Zn^2+^‐containing active site. Similar inhibition profiles have been reported for thiophene‐based sulfonamides and attributed to additional interactions outside the canonical zinc‐binding region (Alım et al. 2020). Likewise, the methyl‐substituted derivative **5f** exhibited only moderate inhibitory activity, whereas the bromo‐substituted analogue **5g** displayed enhanced inhibition relative to **5f**, suggesting that the greater polarizability and hydrophobic character of bromine contribute favorably to enzyme binding.

Finally, the unsubstituted derivatives **5a** and **6a** displayed only moderate inhibitory activity, indicating that the benzazole scaffold alone provides limited affinity for the enzyme. Nevertheless, the approximately twofold higher potency of **6a** relative to **5a** further supports the beneficial contribution of the oxygen‐containing benzoxazolone scaffold to carbonic anhydrase inhibition.

Analysis of the selectivity indices derived from the inhibition constants (*Kᵢ*) demonstrated that all synthesized compounds preferentially inhibited hCA II over hCA I, with SI values ranging from **2.57 to 5.28** (Table 2). Among the evaluated derivatives, **6d** exhibited the highest CA II selectivity (SI = 5.28), followed by **6c** (SI = 4.21), indicating that the combination of an oxygen‐containing benzoxazolone scaffold and electron‐withdrawing substituents is particularly favorable for selective hCA II inhibition. Within the benzothiazolone series, the chloro‐ and bromo‐substituted derivatives **5b** (SI = 3.77) and **5g** (SI = 3.69) also displayed pronounced CA II selectivity, whereas the fluoro‐substituted analogue **5c** (SI = 0.92) exhibited nearly equivalent affinity toward both isoforms.

Comparison of structurally corresponding sulfur‐ and oxygen‐containing analogue pairs (**5b–6b**, **5c–6c**, **5d–6d**, and **5e–6e**) further demonstrated that replacing sulfur with oxygen generally enhanced CA II selectivity, with the most pronounced improvements observed for the fluoro‐ and nitro‐substituted derivatives. Collectively, these findings indicate that CA isoform selectivity is governed by the combined influence of the aromatic substitution pattern and the scaffold heteroatom, with oxygen‐containing analogues bearing electron‐withdrawing substituents providing the most favorable profile for selective hCA II inhibition.

Overall, the present SAR analysis indicates that carbonic anhydrase inhibition is primarily governed by the combined effects of the aromatic substituent and the benzazole scaffold. Oxygen‐containing benzoxazolone derivatives bearing electron‐withdrawing substituents exhibited the highest inhibitory potency and hCA II selectivity, highlighting this scaffold as a promising framework for further optimization. Nevertheless, the proposed structure–activity relationships should be interpreted together with the molecular docking and molecular dynamics analyses presented below.

It should be noted that the selectivity indices were determined using purified enzymes under *in vitro* conditions and therefore reflect differences in binding affinity rather than biological selectivity. Accordingly, complementary cellular and *in vivo* studies are required to confirm whether the observed hCA II selectivity is maintained under physiologically relevant conditions.

#### 2.2.3 α‐Glucosidase Inhibitory Activity and Structure–Activity Relationship (SAR) Studies

**FIGURE 6:**
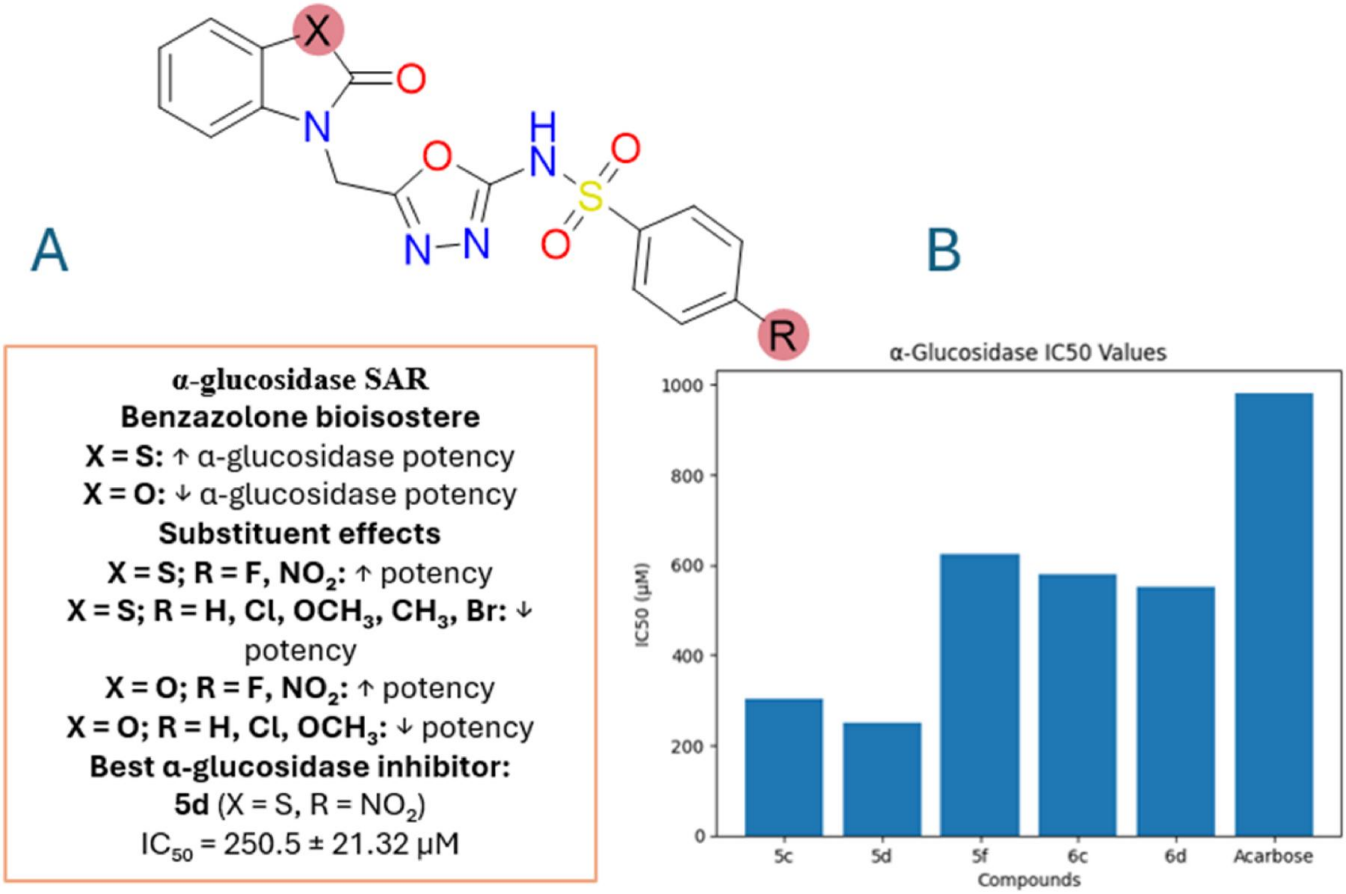
Structure–activity relationship of the synthesized benzothiazolone and benzoxazolone derivatives against α‐glucosidase. (A) Summary of the observed SAR trends according to scaffold and aromatic substitution. (B) Comparison of IC_50_ values for α‐glucosidase inhibition.

The synthesized compounds were evaluated for their inhibitory activity against α‐glucosidase (Table 3, Figure 6). Overall, the series exhibited moderate to weak inhibition compared with their pronounced activities against cholinesterases and carbonic anhydrase isozymes. Only **5c**, **5d**, **5f**, **6c**, and **6d** showed measurable inhibitory activity, whereas the remaining derivatives failed to reach 50% inhibition at the highest concentrations tested because of limited activity and solubility constraints.

**TABLE 3:** Inhibitory activities of the synthesized compounds against α‐glucosidase.

	α‐Glucosidase	
Compounds	IC_50_ (µM)	% Inhibition (400 µM)
**5a**	ND	13.68 ± 1.77
**5b**	ND	14.95 ± 2.76
**5c**	304.03 ± 21.77	56.71 ± 1.76
**5d**	250.5 ± 21.32	66.79 ± 3.97
**5e**	ND	11.20 ± 0.09
**5f**	623.83 ± 7.01	35.42 ± 0.23
**5g**	No activity	No activity
**6a**	ND	13.53 ± 0.74
**6b**	ND	10.46 ± 3.20
**6c**	578.43 ± 15.07	32.78 ± 0.99
**6d**	551.7 ± 26.15	33.78 ± 0.44
**6e**	ND	10.15 ± 0.51
**Acarbose**	981.57 ± 67.34	22.42 ± 3.33

*Note*: ND (Not detected): IC_50_ values were not determined as the compounds failed to reach 50% inhibition at the highest concentrations tested (600 or 800 µM). Evaluation at higher concentrations was not possible due to solubility limitations resulting in precipitation.

A clear scaffold‐dependent SAR was observed. Overall, the benzothiazolone derivatives (series **5**) exhibited stronger α‐glucosidase inhibition than the corresponding benzoxazolone analogues (series **6**). Within both scaffold series, electron‐withdrawing substituents, particularly **NO_2_** and **F**, enhanced inhibitory potency, whereas unsubstituted and electron‐donating analogues generally displayed weak or no activity. Consistent with this trend, **5d** was identified as the most potent α‐glucosidase inhibitor (IC_50_ = 250.5 ± 21.32 µM), followed by **5c** (IC_50_ = 304.03 ± 21.77 µM).

Although **5d** and **5c** exhibited lower IC_50_ values than the reference inhibitor acarbose, the overall inhibitory profile remained considerably weaker than that observed against AChE, BChE, hCA I, and hCA II, suggesting that the present scaffold is better suited for cholinesterase and carbonic anhydrase inhibition. Owing to the limited inhibitory activity of most derivatives, enzyme kinetic studies were not performed for α‐glucosidase.

### 2.3 In Silico Simulations

#### 2.3.1 Molecular Docking

Prior to docking of the synthesized compounds, the docking protocol was validated by redocking the co‐crystallized ligands into the active sites of hCA I (PDB: 6Y00), hCA II (PDB: 6G3Q), AChE (PDB: 4EY6), and BChE (PDB: 4BDS). The predicted binding poses reproduced the key crystallographic interactions reported for ligand 1, famotidine, galantamine, and tacrine, respectively, showing good agreement between the docking and experimental structures (Ali et al. 2020; Angeli et al. 2018a; Cheung et al. 2012; Nachon et al. 2013). Overall, the validated docking protocol successfully identified the reported active‐site regions and interaction patterns of the reference ligands, supporting its suitability for subsequent analysis of the synthesized compounds. In addition, molecular dynamics (MD) simulations were performed to further evaluate the stability of the docked protein–ligand complexes.

To rationalize the cholinesterase inhibitory activities, molecular docking studies were performed using the most active compounds against AChE (**5b**, **5d**, **5e**, **5g**, and **6b**) and BChE (**5b**, **5c**, **5e**, **5g**, **6b**, and **6c**) (Table 4). The selected compounds displayed favorable docking scores and glide energy values toward both enzymes, supporting the results obtained from the enzymatic assays. In general, docking scores of the synthesized compounds were comparable to those of the co‐crystallized ligands galantamine (AChE, PDB: 4EY6) and tacrine (BChE, PDB: 4BDS), whereas their glide energy values were generally lower, suggesting favorable binding interactions.

**TABLE 4:** Docking score, glide energy, interaction types, and residues of active and co‐crystalline compounds in their binding to AChE and BChE structures.

Compound	Target	Docking score	Glide energy	Interaction types and residues	
				H‐bond	Pi‐pi/pi‐ion
**5b**	4EY6	−6.55	−48.67	—	Trp86, Tyr124, Tyr337
**5d**	4EY6	−6.93	−47.16	Gly122, Phe295	Tyr124, Trp286(3), Tyr337, Phe338
**5e**	4EY6	−7.72	−51.54	—	Tyr124, Trp286, Tyr337, Phe338
**5g**	4EY6	−6.31	−48.79	—	Trp86, Phe338
**6b**	4EY6	−7.27	−45.77	Gly121, Gly122, Phe295	Tyr224, Trp286, Tyr337, Phe338
**Galantamine**	4EY6	−9.34	−41.16	Gly122, Tyr133, Glu202, Tyr337	—
**5b**	4BDS	−7.76	−46.79	—	His438
**5c**	4BDS	−7.25	−49.82	Tyr128	His438
**5e**	4BDS	−7.49	−52.41	Tyr128	His438
**5g**	4BDS	−7.52	−51.31	—	His438
**6b**	4BDS	−7.33	−51.84	Gly117	Trp82(x2), His438
**6c**	4BDS	−6.52	−45.52	—	Trp82(x3), His438
**Tacrine**	4BDS	−7.92	−33.82	His438	Trp82(x2)

**FIGURE 7:**
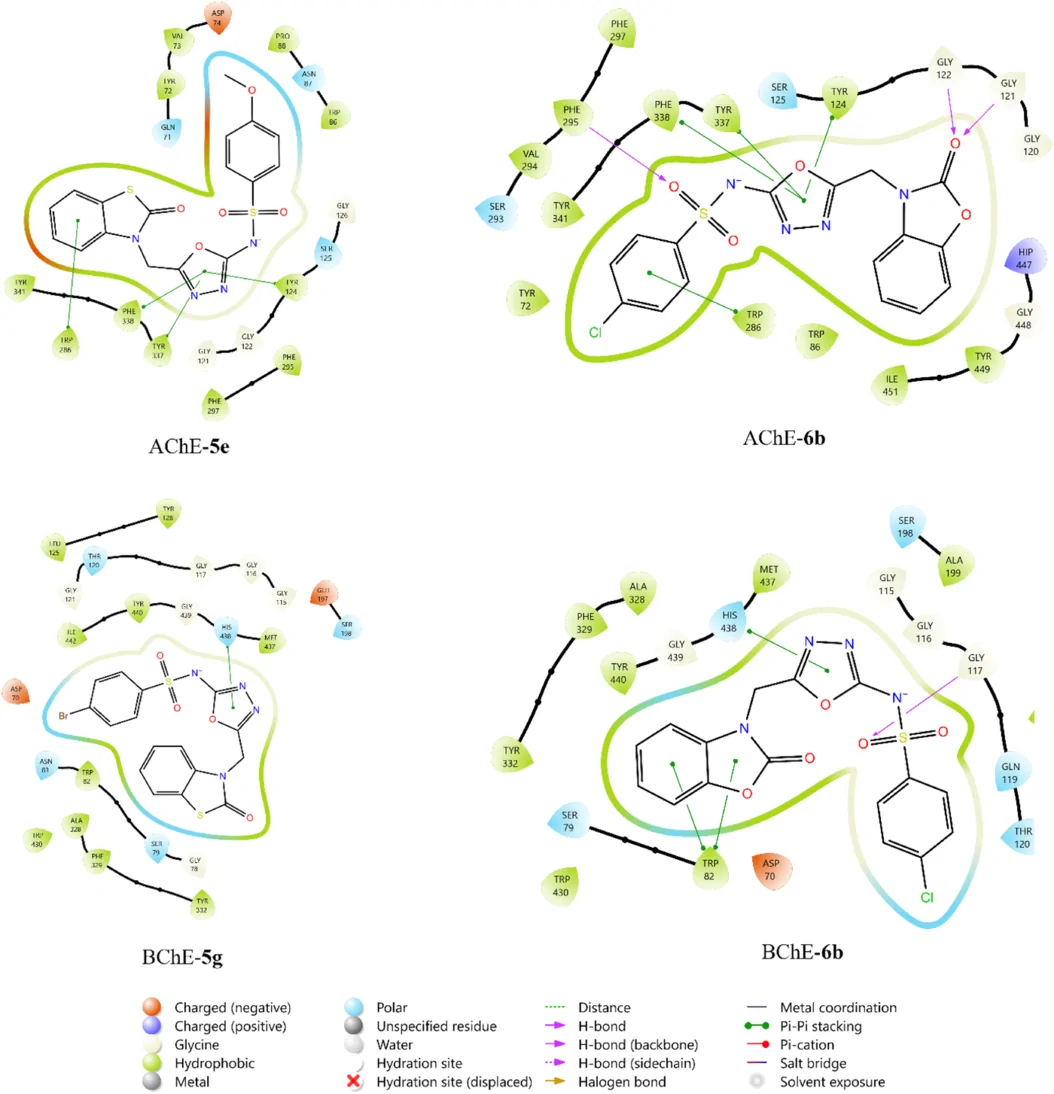
Binding profiles of compounds **5e** and **6b** in their interaction with AChE structure (4EY6) and compounds **5g** and **6b** in their interaction with BChE structure (4BDS).

Within the AChE active site, the compounds established extensive π–π interactions with key aromatic residues, including Trp86, Tyr124, Trp286, Tyr337, and Phe338. Among the investigated derivatives, **6b** exhibited one of the most favorable interaction profiles, forming hydrogen bonds with Gly121, Gly122, and Phe295 in addition to multiple aromatic interactions. These residues are located within or near the catalytic gorge and peripheral anionic site, supporting the experimentally observed competitive inhibition pattern. For visualization, compound **5e** was selected as the representative AChE ligand because it exhibited the most favorable docking score, whereas compound **6b** was chosen because it formed the largest number of hydrogen‐bond interactions (Figure 7).

For BChE, the docked compounds predominantly interacted with Trp82 and His438, residues known to play important roles in ligand recognition and stabilization. The most active BChE inhibitor, **5g**, displayed docking characteristics comparable to those of tacrine, combining a favorable docking score with a substantially lower glide energy value. Similarly, **6b** and **6c** established multiple interactions with Trp82 and His438, supporting their strong inhibitory activities.

Overall, the docking results were consistent with the enzyme inhibition data and confirmed that the synthesized compounds can effectively occupy the active‐site regions of both cholinesterases through a combination of aromatic and hydrogen‐bonding interactions (Figure 7). The potential of the most active compounds to act on the binding pocket of the cholinesterase structures implied competitive binding.

**FIGURE 8:**
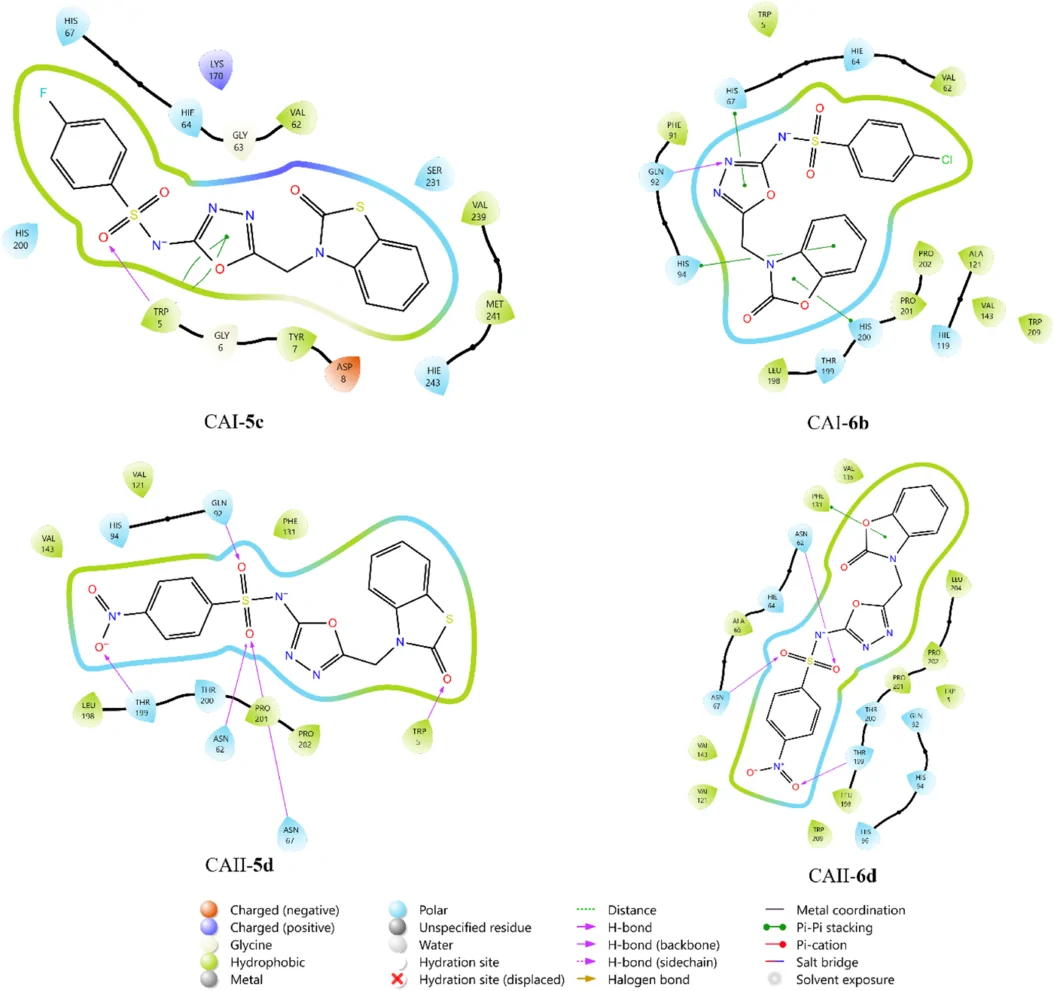
Binding profiles of compounds **5c** and **6b** in their interaction with human CAI structure (6Y00) and compounds **5d** and **6d** in their interaction with human CAII structure (6G3Q).

Molecular docking studies were performed using the most active compounds against hCA I (**5c**, **5d**, **6b**, **6c**, and **6d**) and hCA II (**5b**, **5d**, **6b**, **6c**, and **6d**). All selected compounds occupied the catalytic cavities of both isoenzymes and established interactions with key active‐site residues involved in ligand recognition and stabilization (Table 5, Figure 8).

**TABLE 5:** Docking score, glide energy, interaction types, and residues of relatively active compound as well as co‐crystalline ligands in their binding to human CAI (6Y00) and CAII (6G3Q) structures.

Compound	Target	Docking score	Glide energy	Interaction types and residues		
				H‐bond	Pi‐pi/pi‐ion	Halogen/Salt bridge
**5c**	6Y00	−3.91	−41.60	Trp5	Trp5(x2)	—
**5d**	6Y00	−3.86	−39.91	Lys170	Trp5, His64	—
**6b**	6Y00	−4.44	−35.17	Gln92	His67, His94, His200	—
**6c**	6Y00	−3.25	−38.57	Asn69, Gln92	His64	—
**6d**	6Y00	−3.77	−38.73	Gln92	—	His200
**Ligand 1**	6Y00	−5.32	−35.97	Trp5, Gln92, Tyr204	—	—
**5b**	6G3Q	−4.45	−39.58	Asn67	Phe131	Asn62
**5d**	6G3Q	−4.95	−41.44	Trp5, Asn62, Asn67, Gln92, Thr199	—	—
**6b**	6G3Q	−4.59	−40.11	Trp5	—	—
**6c**	6G3Q	−4.69	−39.42	Gln92	His94	—
**6d**	6G3Q	−4.52	−42.20	Asn62, Asn67, Thr199	Phe131	—
**Ligand 2**	6G3Q	−5.31	−34.07	His94, Thr199, Thr200	—	—

For hCA I, the investigated compounds interacted primarily with residues including Trp5, Gln92, His64, His67, and His200. Among them, **6b** exhibited the most favorable docking score (−4.44) and formed multiple stabilizing interactions, including a hydrogen bond with Gln92. For hCA II, docking scores of the investigated compounds were generally comparable. The most favorable docking profile was observed for 5d (docking score = −4.95), which established multiple hydrogen‐bond interactions involving Trp5, Asn62, Asn67, Gln92, and Thr199. In addition, **6d**, the most potent hCA II inhibitor in the enzymatic assays, formed stabilizing interactions with Asn62, Asn67, Thr199, and Phe131, supporting its strong inhibitory activity.

Overall, the docking results were in good agreement with the enzyme inhibition data. Notably, compounds displaying the strongest inhibitory activities in vitro, particularly **5d** and **6d**, also exhibited favorable docking profiles, supporting the proposed structure–activity relationships and providing a molecular rationale for their carbonic anhydrase inhibitory activities (Figure 8).

#### 2.3.2 MD Simulation

**FIGURE 9:**
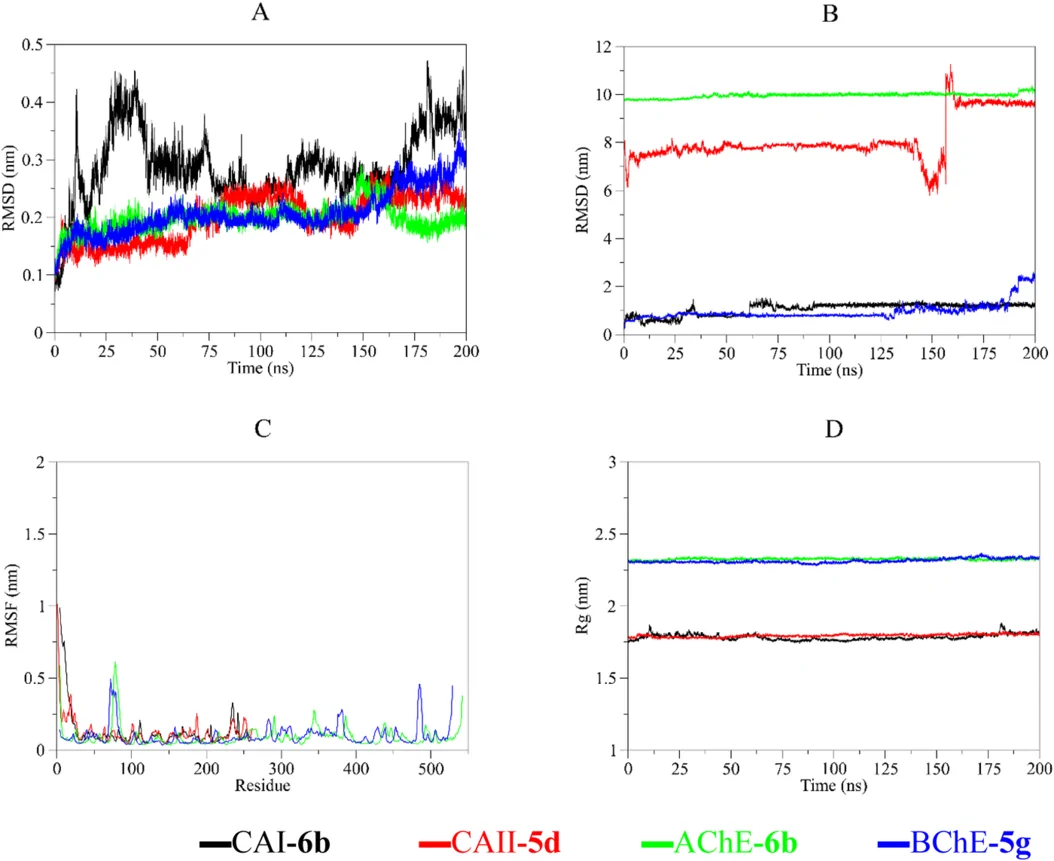
Backbone RMSD (A), compound RMSD (B), RMSF (C), and Rg (D) plots of representative complexes from simulation trajectories. **▬**CAI**−6b ▬**CAII**−5d ▬**AChE**−6b ▬**BChE**−5g**.

Relative stabilities of complexes formed between enzyme structures and compounds with higher binding potential in the docking study were evaluated through MD simulations. To this end, complexes of human CAI with **6b**, CAII with **5d**, AChE with **6b**, and BChE with **5g** were assessed through MD simulation for 200 ns duration. Root mean square deviation (RMSD), root mean square fluctuation (RMSF), and radius of gyration (Rg) plots were sketched for analyzing trajectories from simulation. Backbone RMSD is used to measure general stability of complexes during simulation. RMSD plots of complex structures depicted that stable complexes were formed by most of the compounds in their binding to target enzyme structures. Complex structures with CAII and AChE structures have given steady RMSD values all over the simulation. The complex with the BChE structure has also given steady values in general. Together with this, it has given rising values in narrow time interval between 94 ns and 97 ns. This condition was reverted as RMSD values were decreasing thereafter. The complex with human CAI structure was anticipated to exhibit the lowest stability among the investigated complex structures. Because it has given higher RMSD values including values above 0.3 nm in some intervals with higher changes (Figure 9A). Compound RMSD is used to measure the location of the compounds relative to the center of the structure during simulation (Bahar et al. 2025). Hence, probability of compounds to preserve their position during simulation is measured by sketching compound RMSD plots. RMSD plots of the compounds have given values with diverse ranges. Compound **6b** inside human CAI structure and compound **5g** inside BChE structure have given lower RMSD values that remained within a given range. A mild RMSD rise was observed for compound **6b** in the 186–192 ns interval. Compound **6b** inside the AChE structure has given high RMSD values but is still steady. Hence, compound **6b** would preserve its position, which is expected to be far from the center of the complex, during simulation. On the other hand, compound **5d** inside the human CAII structure was predicted to exhibit the lowest probability to preserve its position. Because it has had remarkable RMSD changes in the 142–160 ns interval that would enforce it to move out (Figure 9B).

RMSF plots are used to measure per residue perturbations of a structure relative to mean reference. The RMSF plots of the four structures remained stable with significant changes in some narrow residual intervals. A significant RMSF value rise was observed for the complex structures except the BChE complex at the N‐terminal end. In addition, complexes of AChE and BChE structures have had higher values in the 70–83 residual intervals. Similarly, RMSF values of the BChE complex have given higher values in the 481–488 residual intervals. A mild rise was observed for the complexes AChE and BChE structures at their C‐terminals (Figure 9C). Rg plots are used to measure compactness of apo enzyme or their complexes during simulation. Rg values of carbonic anhydrase structures were found to be similar with nearly 1.79 nm average. Similarly, Rg values of cholinesterase structures were found to be similar with nearly 2.32 nm average. In general, complex structures have given steady Rg values during simulation implying a preserved compactness for them. Together with this, cholinesterase structures have had higher Rg values than carbonic anhydrase structures implying lower compactness for cholinesterase (Figure 9D). To wrap up, MD simulation study disclosed the formation of stable complexes in the docking study. Together with this, cholinesterase complex structures were anticipated to have higher stability relatively.

Overall, the MD simulation results supported the formation of stable protein–ligand complexes and were consistent with the docking and enzyme inhibition data. In particular, the AChE–**6b**, BChE–**5g**, and hCA II–**5d** complexes maintained favorable interaction profiles throughout the simulations, supporting their strong binding characteristics and the reliability of the proposed binding modes.

#### 2.3.3 In Silico ADMET Predicted Profiles and Drug‐Likeness Evaluation

For evaluating the therapeutic potential and pharmacokinetic safety profile of the synthesized derivatives, the ADMET (Absorption, Distribution, Metabolism, Excretion, and Toxicity) properties of the most active compounds (**5b**, **5g**, **6c**, and **6d**) were predicted using the SwissADME (Daina et al. 2017) and pkCSM (Pires et al. 2015) web‐based servers based on the in vitro enzyme inhibition results (Table 6).

**TABLE 6:** Predicted physicochemical and ADMET properties of selected compounds.

		5b	5 g	6c	6d
Physicochemical Properties Lipophilicity	Formula	C_16_H_11_ClN_4_O_4_S_2_	C_16_H_11_BrN_4_O_4_S_2_	C_16_H_11_FN_4_O_5_S	C_16_H_11_N_5_O_7_S
	MW (g/mol)	422.87	467.32	390.35	417.35
	Rotatable bonds	5	5	5	6
	HBA	6	6	8	9
	HBD	1	1	1	1
	MR	101.81	104.50	91.15	100.01
	TPSA Å^2^	143.71	143.71	128.61	174.43
	consensus logP	2.15	2.23	1.41	0.24
Druglikeness	Lipinski	Yes	Yes	Yes	Yes
	Ghose	Yes	Yes	Yes	Yes
	Veber	No; 1 violation: TPSA > 140	No; 1 violation: TPSA > 140	Yes	No; 1 violation: TPSA > 140
	Egan	No; 1 violation: TPSA > 131.6	No; 1 violation: TPSA > 131.6	Yes	No; 1 violation: TPSA > 131.6
	Muegge	Yes	Yes	Yes	No; 1 violation: TPSA > 150
Absorption	Caco2 permeability (log Papp in 10^−6 ^cm/s)	1.473	1.474	1.432	0.433
	Intestinal absorption (human) % Absorbed	88.621	88.357	86.442	82.973
Distribution	BBB permanent (log BB)	−1.46	−1.468	−1.495	−1.818
	CNS permeability (log PS)	−2.819	−2.796	−3.876	−3.257
Metabolism	CYP2D6 substrate	No	No	No	No
	CYP3A4 substrate	Yes	Yes	Yes	Yes
	CYP1A2 inhibitior	Yes	Yes	Yes	No
	CYP2C19 inhibitior	Yes	Yes	No	No
	CYP2C9 inhibitior	Yes	Yes	Yes	Yes
	CYP2D6 inhibitior	No	No	No	No
	CYP3A4 inhibitior	Yes	Yes	Yes	Yes
Excretion	Total Clearance (log ml/min/kg)	−0.182	−0.203	0.518	0.663
	Renal OCT2 substrate	No	No	No	No
Toxicity	AMES toxicity	No	No	No	Yes
	Max. tolerated dose (human) (log mg/kg/day)	0.648	0.649	0.608	0.406
	hERG I inhibitor	No	No	No	No
	hERG II inhibitor	No	No	No	No
	Oral Rat Acute Toxicity (LD50) (mol/kg)	2.38	2.389	2.332	2.745
	Hepatotoxicity	Yes	Yes	Yes	Yes

*Note:* Lipinski filter: MW ≤ 500; MLOGP ≤ 4.15; HBA ≤ 10; HBD ≤ 5. Ghose filter: 160 ≤ MW ≤ 480; − 0.4 ≤ WLOGP ≤ 5.6; 40 ≤ MR ≤ 130; 20 ≤ atoms ≤ 70. Veber filter: Rotatable bonds ≤ 10; TPSA ≤ 140 Å2. Egan filter: WLOGP ≤ 5.88; TPSA ≤ 131.6. Muegge filter: 200 ≤ MW ≤ 600, − 2 ≤ XLOGP ≤ 5; TPSA ≤ 157; HBA ≤ 10; HBD ≤ 5; RB ≤ 15; Number of rings ≤ 7; Number of carbons > 4; Number of heteroatoms > 1.

An estimated ADMET profile was generated for representative lead compounds (**5b**, **5g**, **6c**, and **6d**), selected based on their superior inhibitory activities against different biological targets in the in vitro enzyme assays.

All selected compounds satisfied the Lipinski and Ghose criteria, whereas **5b**, **5g**, and **6d** violated the Veber and Egan rules because of their relatively high topological polar surface area (TPSA). Despite these violations, all compounds were predicted to exhibit high human intestinal absorption (82.9%–88.6%), indicating acceptable oral absorption potential. Among the evaluated derivatives, **6c** displayed the most balanced physicochemical profile, combining moderate lipophilicity (consensus logP = 1.41), acceptable TPSA (128.61 Å^2^), and compliance with all major drug‐likeness filters (Table 6).

The predicted distribution profiles indicated limited blood–brain barrier (BBB) penetration for all compounds, as reflected by their negative logBB values (−1.46 to −1.82). Likewise, the predicted CNS permeability values suggested restricted central nervous system exposure. Nevertheless, **5b** and **5g** showed relatively more favorable BBB‐ and CNS‐related properties than **6c** and **6d**, which is consistent with their comparatively higher lipophilicity. These results suggest that further structural optimization may be required to enhance BBB permeability and CNS exposure, thereby improving the suitability of these compounds as centrally acting cholinesterase inhibitors (Table 6).

All compounds were predicted to be CYP3A4 substrates and potential CYP3A4 inhibitors, indicating the possibility of metabolic interactions. However, none were predicted to be CYP2D6 substrates or inhibitors. Importantly, no hERG I or hERG II inhibition was predicted for any of the investigated compounds. While **5b**, **5g**, and **6c** were predicted to be non‐mutagenic in the AMES test, **6d** showed a potential AMES toxicity alert. Although **6d** exhibited the most potent hCA II inhibitory activity, its predicted mutagenicity and relatively high polarity may limit its further development as a lead candidate. Future structural optimization could therefore focus on reducing these potential liabilities through appropriate bioisosteric replacement of the nitro group or by introducing alternative electron‐withdrawing substituents while preserving the favorable interactions responsible for carbonic anhydrase inhibition. Such modifications may improve the predicted safety profile without compromising biological activity (Table 6).

Overall, 6c emerged as the compound with the most favorable predicted drug‐like profile, whereas 5b combined potent cholinesterase inhibition with comparatively advantageous CNS‐related properties. In contrast, the excellent enzymatic activity of 6d was partially offset by its high polarity and predicted mutagenicity risk, highlighting the need for further optimization and experimental safety evaluation. It should be noted that these ADMET properties were obtained using predictive computational models and therefore require experimental validation before definitive pharmacokinetic or toxicological conclusions can be drawn.

## 3 Conclusion

A series of 12 novel sulfonamide‐based 2‐amino‐1,3,4‐oxadiazole derivatives incorporating benzothiazolone and benzoxazolone scaffolds was successfully synthesized and evaluated as multifunctional enzyme inhibitors. The synthesized compounds exhibited potent nanomolar inhibition of AChE and BChE together with significant inhibition of hCA I and hCA II, whereas only moderate α‐glucosidase inhibitory activity was observed. Kinetic studies demonstrated competitive inhibition against AChE and predominantly mixed‐type inhibition against BChE, providing further insight into their inhibition mechanisms.

Structure–activity relationship analyses revealed that both the nature of the benzazole scaffold and aromatic substitution substantially influenced enzyme inhibition. Halogen substitution generally enhanced cholinesterase inhibition, with **5b**, **5g**, **6b**, and **6c** emerging as the most active derivatives. Among them, **5b** exhibited the most favorable cholinesterase profile by combining potent AChE inhibition (*Kᵢ* = 7.1 ± 0.6 nM) with the highest AChE selectivity, whereas **5g** was identified as the most potent BChE inhibitor (*Kᵢ* = 8.4 ± 0.8 nM). In contrast, benzoxazolone derivatives generally displayed superior carbonic anhydrase inhibition, and **6d** was identified as the most potent and selective hCA II inhibitor (*Kᵢ* = 0.43 ± 0.037 µM), highlighting this scaffold as particularly promising for carbonic anhydrase‐targeted inhibitor development.

Molecular docking and molecular dynamics simulations complemented the experimental findings by providing a structural rationale for the observed inhibition profiles and supporting the dynamic stability of the representative protein–ligand complexes. The computational analyses revealed favorable interaction networks within the catalytic pockets of the target enzymes and were consistent with the experimentally observed structure–activity relationships.

Integration of the biological data with the predicted ADMET profiles identified **5b** as the most promising cholinesterase‐oriented lead candidate because of its potent inhibitory activity, favorable selectivity, and comparatively advantageous CNS‐related properties. However, the predicted negative logBB values indicate limited blood–brain barrier penetration for the current series, suggesting that further structural optimization will be required to improve CNS exposure for the development of anti‐Alzheimer drug candidates. Meanwhile, **6c** exhibited the most balanced predicted drug‐like profile, whereas the excellent hCA II inhibitory activity of **6d** was accompanied by higher polarity and a predicted AMES mutagenicity alert, indicating that future optimization through appropriate bioisosteric modification may further improve its developability.

Overall, the present study demonstrates that sulfonamide‐linked benzazolone–oxadiazole hybrids represent a promising scaffold for the development of multifunctional enzyme inhibitors. Although the current series was initially designed as multitarget cholinesterase and carbonic anhydrase inhibitors, the combination of superior carbonic anhydrase inhibitory potency, favorable selectivity, and supportive computational findings suggests that these compounds may be particularly attractive as lead structures for the future development of carbonic anhydrase‐targeted therapeutics. Nevertheless, the biological selectivity, pharmacokinetic characteristics, and toxicity profiles reported herein are based primarily on enzyme‐based assays and computational predictions. Therefore, further cell‐based investigations, experimental ADMET studies, pharmacokinetic evaluation, and in vivo validation will be required to confirm the therapeutic potential of these compounds and determine whether the observed activities are maintained under physiologically relevant conditions.

## 4 Experimental Section

### 4.1 Chemical Section

All reagents and solvents were purchased from commercial suppliers and used without further purification. Microwave‐assisted reactions were performed using a Milestone Microwave Organic Synthesis Reactor. Melting points were determined on an SMP‐II melting point apparatus and are uncorrected.

Structural characterization was performed by ^1^H NMR, ^13^C NMR, LC–MS, and HRMS analyses. NMR spectra were recorded in DMSO‐*d*_*6*_ on a Bruker 500 MHz spectrometer using tetramethylsilane (TMS) as the internal standard. Chemical shifts are reported in ppm and coupling patterns are designated as s, d, dd, ddd, t, td, and m.

High‐resolution mass spectra were acquired using a Waters LCT Premier XE TOF mass spectrometer equipped with an ESI(+) source coupled to a UPLC system. Compound purity was assessed by TLC and LC–MS analyses using a C18 reversed‐phase column with water/acetonitrile or methanol mobile phases containing 0.1% formic acid.

The intermediate compounds **1a–b**, **2a–b**, **3a–b**, and **4a–b** were synthesized according to previously reported procedures (Arslan et al. 2023; Arslan Karahan et al. 2025A; Önkol et al. 2012; Shaharyar et al. 2016).

**General synthesis method of N‐(5‐((2‐oxo‐1,3‐benzazol‐3(2** ***H*** **)‐yl)methyl)−1,3,4‐oxadiazol‐2‐yl)benzenesulfonamide derivative compounds (5a–g, 6a–e):** The appropriate intermediate (**4a** or **4b**, 1.0 equiv) was dissolved in chloroform and treated with pyridine (1.5 equiv) and the corresponding benzenesulfonyl chloride derivative (2.5 equiv). The reaction mixture was stirred at room temperature for 12 h and then extracted with saturated aqueous Na_2_CO_3_ solution. The organic layer was separated, concentrated under reduced pressure, and the resulting crude product was recrystallized from ethanol to afford the target benzenesulfonamide derivatives (**5a–g** and **6a–e**). The relatively low isolated yields of compounds **5f** and **5g** were mainly attributed to pronounced oiling‐out of the crude products during the work‐up process. Although slight oiling‐out was also observed for several other derivatives, these products readily solidified upon standing or cooling, whereas compounds **5f** and **5g** remained oily for a prolonged period, making isolation and purification more difficult and resulting in greater product loss.

***N*** **‐(5‐((2‐Oxobenzo[** ***d*** **]thiazol‐3(2** ***H*** **)‐yl)methyl)−1,3,4‐oxadiazol‐2‐yl)benzenesulfonamide (5a)**

Yield: % 47; MP: 156°C

HRMS (m/z) [M + H]^+^ for C_16_H_12_N_4_O_4_S_2_ calculated: 389.0378 found: 389.0363

^1^H NMR (500 MHz, DMSO) δ = 7.83 (d, *J* = 7.2, 2H), 7.72 (d, *J* = 7.8, 1H), 7.60 (t, *J* = 7.4, 1H), 7.52 (t, *J* = 7.7, 2H), 7.47 − 7.36 (m, 2H), 7.28 − 7.24 (m, 1H), 5.31 (s, 2H).

^13^C NMR (125 MHz, DMSO) δ 169.38, 156.70, 154.32, 142.42, 136.50, 132.87, 129.46, 127.27, 126.52, 124.27, 123.59, 121.64, 112.08, 37.28.

**4‐Chloro‐** ***N*** **‐(5‐((2‐oxobenzo[** ***d*** **]thiazol‐3(2** ***H*** **)‐yl)methyl)−1,3,4‐oxadiazol‐2‐yl)benzenesulfonamide (5b)**

Yield: % 53; MP: 191°C

HRMS (m/z) [M + H]^+^ for C_16_H_11_ClN_4_O_4_S_2_ calculated: 422.9989 found: 422.9991

^1^H NMR (500 MHz, DMSO) δ = 7.87 − 7.77 (m, 2H), 7.72 (d, *J* = 8.0, 1H), 7.60 − 7.52 (m, 2H), 7.44 − 7.37 (m, 2H), 7.29 − 7.23 (m, 1H), 5.31 (s, 2H).

^13^C NMR (125 MHz, DMSO) δ 169.37, 156.93, 154.44, 141.38, 137.60, 136.50, 129.53, 128.56, 127.26, 124.27, 123.59, 121.63, 112.09, 37.26.

**4‐Fluoro‐** ***N*** **‐(5‐((2‐oxobenzo[** ***d*** **]thiazol‐3(2** ***H*** **)‐yl)methyl)−1,3,4‐oxadiazol‐2‐yl)benzenesulfonamide (5c)**

Yield: % 44; MP: 182°C

HRMS (m/z) [M + H]^+^ for C_16_H_11_FN_4_O_4_S_2_ calculated: 407.0284 found: 407.0274

^1^H NMR (500 MHz, DMSO) δ = 7.91 − 7.86 (m, 2H), 7.72 (d, *J* = 8.7, 1H), 7.40 (d, *J* = 5.0, 2H), 7.37 − 7.31 (m, 2H), 7.28 − 7.23 (m, 1H), 5.32 (s, 2H).

^13^C NMR (125 MHz, DMSO) δ 169.37, 164.51 (d,^*1*^ *J*_*C‐F*_ = 250.6 Hz), 156.80, 154.38, 138.91 (d,^*4*^ *J*_*C‐F*_ = 2.9 Hz), 136.50, 129.58 (d,^*3*^ *J*_*C‐F*_ = 9.4 Hz), 127.26, 124.27, 123.59, 121.63, 116.52 (d,^*3*^ *J*_*C‐F*_ = 22.7 Hz), 112.08, 37.26.

**4‐Nitro‐** ***N*** **‐(5‐((2‐oxobenzo[** ***d*** **]thiazol‐3(2** ***H*** **)‐yl)methyl)−1,3,4‐oxadiazol‐2‐yl)benzenesulfonamide (5d)**

Yield: % 59; MP: 197°C

HRMS (m/z) [M + H]^+^ for C_16_H_11_N_5_O_6_S_2_ calculated: 434.0229 found: 434.0222

^1^H NMR (500 MHz, DMSO) δ = 8.30 (d, *J* = 8.5, 2H), 8.06 (d, *J* = 8.5, 2H), 7.70 (d, *J* = 7.8, 1H), 7.41 − 7.38 (m, 2H), 7.29 – 7.22 (m, 1H), 5.31 (s, 2H).

^13^C NMR (125 MHz, DMSO) δ 169.35, 157.21, 154.65, 149.83, 147.97, 136.48, 128.22, 127.25, 124.75, 124.25, 123.55, 121.61, 112.09, 37.23.

**4‐Methoxy‐** ***N*** **‐(5‐((2‐oxobenzo[** ***d*** **]thiazol‐3(2** ***H*** **)‐yl)methyl)−1,3,4‐oxadiazol‐2‐yl)benzenesulfonamide (5e)**

Yield: % 42; MP: 152°C

HRMS (m/z) [M + H]^+^ for C_17_H_14_N_4_O_5_S_2_ calculated: 419.0484 found: 419.0489

^1^H NMR (500 MHz, DMSO) δ = 7.79 − 7.74 (m, 2H), 7.72 (d, *J* = 7.7, 1H), 7.43 − 7.35 (m, 2H), 7.28 − 7.23 (m, 1H), 7.05 – 6.96 (m, 2H), 5.31 (s, 2H), 3.81 (s, 3H).

^13^C NMR (125 MHz, DMSO) δ 169.38, 162.61, 156.52, 154.24, 136.50, 134.19, 128.73, 127.28, 124.28, 123.59, 121.64, 114.54, 112.06, 56.08, 37.28.

**4‐Methyl‐** ***N*** **‐(5‐((2‐oxobenzo[** ***d*** **]thiazol‐3(2** ***H*** **)‐yl)methyl)−1,3,4‐oxadiazol‐2‐yl)benzenesulfonamide (5f)**

Yield: % 32; MP: 158°C

HRMS (m/z) [M + H]^+^ for C_17_H_14_N_4_O_4_S_2_ için; calculated: 403.0535 found: 403.0551

^1^H NMR (500 MHz, DMSO) δ = 7.73‐7.69 (m, 3H), 7.40 (d, *J* = 4.2, 2H), 7.30 (d, *J* = 8.0, 2H), 7.27‐7.24 (m, 1H), 5.31 (s, 2H), 2.35 (s, 3H).

^13^C NMR (125 MHz, DMSO) δ 169.37, 156.60, 154.28, 143.11, 139.60, 136.51, 129.85, 127.27, 126.59, 124.26, 123.59, 121.64, 112.06, 37.29, 21.43.

**4‐Bromo‐** ***N*** **‐(5‐((2‐oxobenzo[** ***d*** **]thiazol‐3(2** ***H*** **)‐yl)methyl)−1,3,4‐oxadiazol‐2‐yl)benzenesulfonamide (5g)**

Yield: % 27; MP: 190°C

HRMS (m/z) [M + H]^+^ for C_16_H_11_BrN_4_O_4_S_2_ calculated: 466.9483 found: 466.9466

^1^H NMR (500 MHz, DMSO) δ = 7.78 – 7.67 (m, 5H), 7.42 – 7.36 (m, 2H), 7.30 – 7.22 (m, 1H), 5.31 (s, 2H).

^13^C NMR (125 MHz, DMSO) δ 169.36, 156.86, 154.44, 141.77, 136.50, 132.47, 128.66, 127.26, 126.57, 124.27, 123.58, 121.63, 112.10, 37.26.

***N*** **‐(5‐((2‐Oxobenzo[** ***d*** **]oxazol‐3(2** ***H*** **)‐yl)methyl)−1,3,4‐oxadiazol‐2‐yl)benzenesulfonamide (6a)**

Yield: % 39; MP: 161°C

HRMS (m/z) [M + H]^+^ for C_16_H_12_N_4_O_5_S calculated: 373.0607 found: 373.0600

^1^H NMR (500 MHz, DMSO) δ = 7.87 − 7.80 (m, 2H), 7.64 − 7.57 (m, 1H), 7.56 − 7.48 (m, 2H), 7.40 (dd, *J* = 7.8, 1.2, 1H), 7.33 (dd, *J* = 7.8, 1.4, 1H), 7.26 (td, *J* = 7.8, 1.2, 1H), 7.19 (td, *J* = 7.8, 1.4, 1H), 5.20 (s, 2H).

^13^C NMR (125 MHz, DMSO) δ 156.74, 154.22, 153.68, 142.42, 132.88, 130.71, 129.45, 126.51, 124.57, 123.38, 110.38, 110.15, 37.03.

**4‐Chloro‐** ***N*** **‐(5‐((2‐oxobenzo[** ***d*** **]oxazol‐3(2** ***H*** **)‐yl)methyl)−1,3,4‐oxadiazol‐2‐yl)benzenesulfonamide (6b)**

Yield: % 57; MP: 211°C

HRMS (m/z) [M + H]^+^ for C_16_H_11_ClN_4_O_5_S calculated: 407.0217found: 407.0233

^1^H NMR (500 MHz, DMSO) δ = 7.87 − 7.79 (m, 2H), 7.60 − 7.52 (m, 2H), 7.40 (dd, *J* = 7.9, 1.2, 1H), 7.33 (dd, *J* = 7.9, 1.4, 1H), 7.26 (td, *J* = 7.8, 1.2, 1H), 7.19 (td, *J* = 7.8, 1.4, 1H), 5.20 (s, 2H).

^13^C NMR (125 MHz, DMSO) δ 157.00, 154.34, 153.68, 142.41, 141.39, 137.61, 130.71, 129.52, 128.57, 124.57, 123.38, 110.37, 110.16, 37.01.

**4‐Fluoro‐** ***N*** **‐(5‐((2‐oxobenzo[** ***d*** **]oxazol‐3(2** ***H*** **)‐yl)methyl)−1,3,4‐oxadiazol‐2‐yl)benzenesulfonamide (6c)**

Yield: % 46; MP: 167°C

HRMS (m/z) [M + H]^+^ for C_16_H_11_FN_4_O_5_S calculated: 391.0512 found: 391.0508

^1^H NMR (500 MHz, DMSO) δ = 7.94 − 7.85 (m, 2H), 7.40 (dd, *J* = 7.9, 1.2, 1H), 7.36‐7.32 (m, 3H), 7.26 (td, *J* = 7.8, 1.2, 1H), 7.19 (td, *J* = 7.8, 1.4, 1H), 5.20 (s, 2H).

^13^C NMR (125 MHz, DMSO) δ 164.51 (d,^*1*^ *J*_*C‐F*_ = 250.8), 156.91, 154.27, 153.68, 142.41, 138.95 (d,^*4*^ *J*_*C‐F*_ = 3.2 Hz), 130.71, 129.59 (d,^*3*^ *J*_*C‐F*_ = 9.5 Hz), 124.57, 123.38, 116.50 (d,^*2*^ *J*_*C‐F*_ = 22.7 Hz), 110.37, 110.15, 37.02.

**4‐Nitro‐** ***N*** **‐(5‐((2‐oxobenzo[** ***d*** **]oxazol‐3(2** ***H*** **)‐yl)methyl)−1,3,4‐oxadiazol‐2‐yl)benzenesulfonamide (6d)**

Yield: % 51; MP: 218°C

HRMS (m/z) [M + H]^+^ for C_16_H_11_N_5_O_7_S calculated: 418.0457 found: 418.0460

^1^H NMR (500 MHz, DMSO) δ = 8.30 (d, *J* = 8.5, 2H), 8.07 (d, *J* = 8.5, 2H), 7.38 (d, *J* = 7.8, 1H), 7.31 (d, *J* = 7.8, 1H), 7.25 (t, *J* = 7.8, 1H), 7.18 (t, *J* = 7.8, 1H), 5.20 (s, 2H).

^13^C NMR (125 MHz, DMSO) δ 157.52, 154.55, 153.66, 149.79, 148.10, 142.38, 130.70, 128.24, 124.72, 124.55, 123.35, 110.32, 110.16, 37.00.

**4‐Methoxy‐** ***N*** **‐(5‐((2‐oxobenzo[** ***d*** **]oxazol‐3(2** ***H*** **)‐yl)methyl)−1,3,4‐oxadiazol‐2‐yl)benzenesulfonamide (6e)**

Yield: % 41; MP: 175°C

HRMS (m/z) [M + H]^+^ for C_17_H_14_N_4_O_6_S calculated: 403.0712 found: 403.0708

^1^H NMR (500 MHz, DMSO) δ = 7.80 − 7.73 (m, 2H), 7.40 (dd, *J* = 7.8, 1.2, 1H), 7.33 (dd, *J* = 7.8, 1.4, 1H), 7.26 (td, *J* = 7.8, 1.2, 1H), 7.19 (td, *J* = 7.8, 1.4, 1H), 7.05 − 6.96 (m, 2H), 5.20 (s, 2H), 3.81 (s, 3H).

^13^C NMR (125 MHz, DMSO) δ 162.60, 156.63, 154.13, 153.68, 142.41, 134.24, 130.71, 128.73, 124.58, 123.39, 114.52, 110.38, 110.13, 56.08, 37.03.

### 4.2 Biological Section

#### 4.2.1 Cholinesterase (AChE and BChE) Inhibition Assay

AChE and BChE inhibitory activities were determined spectrophotometrically according to Ellman's colorimetric method (Ellman et al. 1961). Electric eel AChE (Type VI‐S, EC 3.1.1.7, Sigma) and horse serum BChE (EC 3.1.1.8, Sigma) were used as enzyme sources, whereas acetylthiocholine iodide (1.5 mM) and butyrylthiocholine iodide (1.5 mM) were used as the respective substrates. Assays were performed in 96‐well microplates using 0.1 M phosphate buffer (pH 8.0) and 1.0 mM 5,5′‐dithiobis‐(2‐nitrobenzoic acid) (DTNB). Following pre‐incubation of the enzyme with the test compounds at 25°C for 10 min, the reaction was initiated by the addition of the corresponding substrate. The increase in absorbance resulting from the formation of 5‐thio‐2‐nitrobenzoate was monitored at 412 nm using a BioTek microplate reader. Galantamine and rivastigmine were used as reference inhibitors. All assays were performed in triplicate.

#### 4.2.2 Carbonic Anhydrase I and II (CA I and II) Inhibition Assay

The inhibitory activities of the synthesized compounds against hCA I and hCA II were evaluated using enzymes purified from human erythrocytes by Sepharose‐4B–L‐tyrosine–sulfanilamide affinity chromatography according to the reported procedure (Ozensoy et al. 2004). Human erythrocytes were obtained from anonymized residual blood samples supplied by a local blood bank/hospital blood center. No blood was collected specifically for this study, and no identifiable personal information was accessed. According to institutional regulations, the use of anonymized residual blood materials for enzyme purification studies is exempt from ethical committee approval and informed consent requirements. Enzyme purity was confirmed by SDS–PAGE.

Esterase inhibition activities of the synthesized compounds toward hCA I and hCA II were determined spectrophotometrically using *p*‐nitrophenyl acetate (NPA) as the substrate according to the reported methods (Kasımoğulları et al. 2010; Verpoorte et al. 1967). Assays were performed at 25°C, and the hydrolysis of NPA was monitored at 348 nm for 3 min. Acetazolamide was used as the reference inhibitor. Each compound was evaluated at serial concentrations to generate inhibition curves, and all assays were performed in triplicate.

#### 4.2.3 α‐Glucosidase Inhibition Assay

α‐Glucosidase inhibitory activity was evaluated using a spectrophotometric microplate assay against α‐glucosidase from *Saccharomyces cerevisiae* as previously described. Assays were carried out in 50 mM phosphate buffer (pH 6.8) containing 100 mM NaCl. After preincubation of enzyme and test compounds at 37°C, the reaction was initiated by addition of substrate and monitored at 405 nm. Acarbose was used as the reference inhibitor. Percentage inhibition and IC_50_ values were calculated from concentration–response curves. All measurements were performed in triplicate (Ayan et al. 2021).

#### 4.2.4 Determination of IC_50_ Values

The IC_50_ values of the newly synthesized compounds against AChE, BChE, CA I, and CA II were determined using the corresponding enzyme activity assays described above. Enzyme activity measured in the absence of inhibitors was defined as 100% activity. Residual activities at various inhibitor concentrations were calculated and expressed as percentage activity.

Dose–response curves (% activity vs. inhibitor concentration) were generated using GraphPad Prism 8.0, and IC_50_ values, defined as the inhibitor concentration causing 50% reduction in enzyme activity, were calculated by nonlinear regression analysis.

#### 4.2.5 Determination of Kᵢ Values and Inhibition Type

*Kᵢ* values and inhibition mechanisms of the selected compounds were determined by measuring initial enzyme velocities at different substrate concentrations in the absence and presence of two fixed inhibitor concentrations. Lineweaver–Burk double‐reciprocal plots of 1/V versus 1/[S] were generated using SigmaPlot 8.0. The inhibitor‐free assay represented the control line (I_0_), whereas assays conducted at two inhibitor concentrations generated the I_1_ and I_2_ lines. *Kᵢ* values and inhibition types were determined from the resulting plots according to standard enzyme kinetic procedures (Karataş et al. 2016).

#### 4.2.6 Statistical Analysis

All experiments were performed in triplicate, and the results are expressed as mean ± standard deviation (SD) of three replicate measurements. Descriptive statistical analyses were performed using GraphPad Prism 8.0 (GraphPad Software, San Diego, CA, USA).

### 4.3 In Silico Section

#### 4.3.1 Molecular Docking

Crystal structures of hCAI, hCAII, AChE and BChE were downloaded from the protein data bank (PDB). Human CAI structure with PDB code of 6Y00 and human CAII structure with PDB code of 6G3Q have carried co‐crystalline ligands (Ali et al. 2020; Angeli et al. 2018b). The AChE structure (PDB ID: 4EY6) and the BChE structure (PDB ID: 4BDS) contained co‐crystallized galantamine and tacrine, respectively (Cheung et al. 2012; Nachon et al. 2013). The co‐crystalline inhibitor ligands were used for validating docking procedures and as benchmark standards. Compound structures were sketched through the ChemSketch program. The compound structures were then prepared for the docking by using LigPrep module of Maestro 14.5 program. The possible states of the compounds were generated at pH of 7.00 ± 0.50 with Epik. The enzyme structures were prepared for the docking by using the protein preparation module of the Maestro program. Addition of missing side chains and deleting water were executed in the protein preparation. In addition, optimization and minimization were performed by using OPLS_2005 force field. Protonation states and charges were assigned to the enzyme structures. Enzyme structures’ grid boxes were delimited by using receptor grid generation module of the program. The docking procedure was performed by using Glide docking module of Schrödinger suite release 2025‐3 (Anil et al. 2022).

#### 4.3.2 MD Simulation

Complexes of compounds with the highest binding potential to enzyme structures were selected for further analysis. Relative stabilities of complexes formed with the selected compounds were assessed through MD simulation study. Compound topologies were built through the CGenFF server (Vanommeslaeghe and MacKerell 2012). Enzyme structure topologies were built through GROMACS via the related command line. Thereafter, the complex structures were made ready for the simulation by applying consecutive GROMACS command lines. MD simulation of 200 ns length was performed after the simulation system requirements were met based on the reported studies. MD simulation was undertaken by using GROMACS 2024 (Abraham et al. 2015; Cihan et al. 2025). In the last step, simulation plots were sketched via QtGrace for analyzing the resulting trajectories.

#### 4.3.3 ADMET and Drug‐Likeness Prediction

The physicochemical properties, drug‐likeness, and pharmacokinetic profiles of the synthesized compounds were evaluated using in silico approaches. Drug‐likeness and physicochemical descriptors, including molecular weight, lipophilicity (logP), hydrogen bond acceptors/donors, topological polar surface area (TPSA), were predicted using the SwissADME web server (https://www.swissadme.ch) (Daina et al. 2017).

To further assess the absorption, distribution, metabolism, excretion, and toxicity (ADMET) properties, the pkCSM online tool (https://biosig.lab.uq.edu.au/pkcsm) was employed. This platform utilizes graph‐based signatures to predict pharmacokinetic and toxicity endpoints based on molecular structure (Pires et al. 2015).

All calculations were performed using canonical SMILES representations of the synthesized compounds, which were generated and verified prior to submission into the respective web‐based servers. The obtained data were analyzed comparatively to identify compounds with favorable drug‐likeness and pharmacokinetic profiles.

## Author Contributions

**Gülnur Arslan Karahan:** conceptualization, data curation, formal analysis, funding acquisition, investigation, methodology, project administration, resources, software, supervision, validation, visualization, writing – original draft, writing – review and editing. **Başak Gökçe:** investigation, methodology, validation, visualization, writing – original draft. **Muhammed Tilahun Muhammed:** formal analysis, methodology, software validation, visualization, writing – original draft. **Emre Kadir Ayan:** investigation, methodology, validation, visualization, writing – original draft. **Azime Berna Özçelik:** supervision, investigation, methodology, validation, visualization, writing – original draft.

## Conflicts of Interest

The authors declare no conflicts of interest.

## Supporting information


Supporting File

## Data Availability

The data that support the findings of this study are available in the supplementary material of this article.
