# Supplementary material for: Sulfonamide‐Based 2‐Amino‐1,3,4‐Oxadiazole Derivatives as Dual Cholinesterase and Carbonic Anhydrase Inhibitors: Design, Synthesis, Kinetic Characterization, Molecular Modeling, and ADMET Evaluation
Source: Drug Dev Res. 2026 Aug 25;87(6):e70375. doi: 10.1002/ddr.70375 (PMC13504408; doi:10.1002/ddr.70375)
Supplement: Supplementary file 1 [file DDR-87-e70375-s001.docx]

**Supplementary Data**

**Sulfonamide-Based 2-Amino-1,3,4-Oxadiazole Derivatives as Dual Cholinesterase and Carbonic Anhydrase Inhibitors: Design, Synthesis, Kinetic Characterization, Molecular Modeling, and ADMET Evaluation**

Gülnur Arslan Karahan^1^, Başak Gökçe^2^, Muhammed Tilahun Muhammed^1^, Emre Kadir Ayan^1^, Azime Berna Özçelik^3^

^1^ Department of Pharmaceutical Chemistry Faculty of Pharmacy Suleyman Demirel University, Isparta, 32260, Türkiye

^2^ Department of Biochemistry Faculty of Pharmacy Suleyman Demirel University, Isparta, 32260, Türkiye

^3^ Department of Pharmaceutical Chemistry Faculty of Pharmacy, Gazi University, Ankara, Türkiye

Corresponding author: Gülnur Arslan Karahan

Email: gulnurarslan@sdu.edu.tr

**Table of Contents**

[**1.**	**Chemistry**	3](#_Toc232808190)

[**2.**	**Biological**	27](#_Toc232808191)

[**2.1.**	**Cholinesterase inhibitory activity**	27](#_Toc232808192)

[**2.2.**	**Carbonic Anhydrase isozymes CA I and CA II inhibitory activity**	31](#_Toc232808193)

[**2.3.**	**α-Glucosidase inhibitory activity**	35](#_Toc232808194)

[**3.**	***In silico***	36](#_Toc232808195)

**Table of Figures**

Figure S 1. LC-MS, ^1^H-NMR and ^13^C-NMR spectra of **5a**	4

Figure S 2. LC-MS, ^1^H-NMR and ^13^C-NMR spectra of **5b**	6

Figure S 3. LC-MS, ^1^H-NMR and ^13^C-NMR spectra of **5c**	8

Figure S 4. LC-MS, ^1^H-NMR and ^13^C-NMR spectra of **5d**	10

Figure S 5. LC-MS, ^1^H-NMR and ^13^C-NMR spectra of **5e**	12

Figure S 6. LC-MS, ^1^H-NMR and ^13^C-NMR spectra of **5f**	14

Figure S 7. LC-MS, ^1^H-NMR and ^13^C-NMR spectra of **5g**	16

Figure S 8. LC-MS, ^1^H-NMR and ^13^C-NMR spectra of **6a**	18

Figure S 9. LC-MS, ^1^H-NMR and ^13^C-NMR spectra of **6b**	20

Figure S 10. LC-MS, ^1^H-NMR and ^13^C-NMR spectra of **6c**	22

Figure S 11.LC-MS, ^1^H-NMR and ^13^C-NMR spectra of **6d**	24

Figure S 12. LC-MS, ^1^H-NMR and ^13^C-NMR spectra of **6e**	26

Figure S 13. Dose–response curves of AChE and BChE inhibition	30

Figure S 14. Dose–response curves of hCA I and hCA II inhibition	34

Figure S 15. Dose–response curves of α-glucosidase inhibition	35

Figure S 16. Binding profiles of co-crystalline ligands inside human CAI structure (ligand 1) and human CAII structure (ligand 2)	36

Figure S 17.Binding profiles of compounds **5d**, **6c** and **6d** in their interaction with human CAI structure	37

Figure S 18. Binding profiles of compounds **5b**, **6b** and **6c** in their interaction with human CAII structure	38

Figure S 19. Binding profile of galantamine in its interaction with AChE structure and tacrine in its interaction with BChE structure	39

Figure S 20. Binding profiles of compounds **5b,** **5d**, and **5g** in their interaction with AChE structure	40

Figure S 21. Binding profiles of compounds **5b**, **5c**, **5e** and **6c** in their interaction with BChE structure	41

# 1 **Chemistry**

***N*-(5-((2-Oxobenzo[*d*]thiazol-3(2*H*)-yl)methyl)-1,3,4-oxadiazol-2-yl)benzenesulfonamide (5a)**

Figure S 1. LC-MS, ^1^H-NMR and ^13^C-NMR spectra of **5a**

**4-Chloro-*N*-(5-((2-oxobenzo[*d*]thiazol-3(2*H*)-yl)methyl)-1,3,4-oxadiazol-2-yl)benzenesulfonamide (5b)**

Figure S 2. LC-MS, ^1^H-NMR and ^13^C-NMR spectra of **5b**

**4-Fluoro-*N*-(5-((2-oxobenzo[*d*]thiazol-3(2*H*)-yl)methyl)-1,3,4-oxadiazol-2-yl)benzenesulfonamide (5c)**

Figure S 3. LC-MS, ^1^H-NMR and ^13^C-NMR spectra of **5c**

**4-Nitro-*N*-(5-((2-oxobenzo[*d*]thiazol-3(2*H*)-yl)methyl)-1,3,4-oxadiazol-2-yl)benzenesulfonamide (5d)**

Figure S 4. LC-MS, ^1^H-NMR and ^13^C-NMR spectra of **5d**

**4-Methoxy-*N*-(5-((2-oxobenzo[*d*]thiazol-3(2*H*)-yl)methyl)-1,3,4-oxadiazol-2-yl)benzenesulfonamide (5e)**

Figure S 5. LC-MS, ^1^H-NMR and ^13^C-NMR spectra of **5e**

**4-Methyl-*N*-(5-((2-oxobenzo[*d*]thiazol-3(2*H*)-yl)methyl)-1,3,4-oxadiazol-2-yl)benzenesulfonamide (5f)**

Figure S 6. LC-MS, ^1^H-NMR and ^13^C-NMR spectra of **5f**

**4-Bromo-*N*-(5-((2-oxobenzo[*d*]thiazol-3(2*H*)-yl)methyl)-1,3,4-oxadiazol-2-yl)benzenesulfonamide (5g)**

Figure S 7. LC-MS, ^1^H-NMR and ^13^C-NMR spectra of **5g**

***N*-(5-((2-Oxobenzo[*d*]oxazol-3(2*H*)-yl)methyl)-1,3,4-oxadiazol-2-yl)benzenesulfonamide (6a)**

Figure S 8. LC-MS, ^1^H-NMR and ^13^C-NMR spectra of **6a**

**4-Chloro-*N*-(5-((2-oxobenzo[*d*]oxazol-3(2*H*)-yl)methyl)-1,3,4-oxadiazol-2-yl)benzenesulfonamide (6b)**

Figure S 9. LC-MS, ^1^H-NMR and ^13^C-NMR spectra of **6b**

**4-Fluoro-*N*-(5-((2-oxobenzo[*d*]oxazol-3(2*H*)-yl)methyl)-1,3,4-oxadiazol-2-yl)benzenesulfonamide (6c)**

Figure S 10. LC-MS, ^1^H-NMR and ^13^C-NMR spectra of **6c**

**4-Nitro-*N*-(5-((2-oxobenzo[*d*]oxazol-3(2*H*)-yl)methyl)-1,3,4-oxadiazol-2-yl)benzenesulfonamide (6d)**

Figure S 11.LC-MS, ^1^H-NMR and ^13^C-NMR spectra of **6d**

**4-Methoxy-*N*-(5-((2-oxobenzo[*d*]oxazol-3(2*H*)-yl)methyl)-1,3,4-oxadiazol-2-yl)benzenesulfonamide (6e)**

Figure S 12. LC-MS, ^1^H-NMR and ^13^C-NMR spectra of **6e**

# 2 **Biological**

## 2.1 **Cholinesterase inhibitory activity**

Figure S 13. Dose–response curves of AChE and BChE inhibition

## 2.2 **Carbonic Anhydrase isozymes CA I and CA II inhibitory activity**

Figure S 14. Dose–response curves of hCA I and hCA II inhibition

## 2.3 **α-Glucosidase inhibitory activity**

Figure S 15. Dose–response curves of α-glucosidase inhibition

# 3 ***In silico***

Figure S 16. Binding profiles of co-crystalline ligands inside human CAI structure (ligand 1) and human CAII structure (ligand 2)

Figure S 17.Binding profiles of compounds **5d**, **6c** and **6d** in their interaction with human CAI structure

Figure S 18. Binding profiles of compounds **5b**, **6b** and **6c** in their interaction with human CAII structure

Figure S 19. Binding profile of galantamine in its interaction with AChE structure and tacrine in its interaction with BChE structure

Figure S 20. Binding profiles of compounds **5b,** **5d**, and **5g** in their interaction with AChE structure

Figure S 21. Binding profiles of compounds **5b**, **5c**, **5e** and **6c** in their interaction with BChE structure
